# Supplementary material for: Diffusion MRI Indices and Their Relation to Cognitive Impairment in Brain Aging: The Updated Multi-protocol Approach in ADNI3
Source: Front Neuroinform. 2019 Feb 19;13:2. doi: 10.3389/fninf.2019.00002 (PMC6390411; doi:10.3389/fninf.2019.00002)
Supplement: Supplementary file 1 [file Presentation_1.pdf]

Supplementary Material

Diffusion MRI Indices and their Relation to Cognitive Impairment in Brain Aging: The Updated Multi-protocol Approach in ADNI3

Artemis Zavaliangos-Petropulu*, Talia M. Nir*, Sophia I. Thomopoulos, Robert I. Reid, Matt A. Bernstein, Bret Borowski, Clifford R. Jack, Jr., Michael W. Weiner, Neda Jahanshad, Paul M. Thompson, and the Alzheimer's Disease Neuroimaging Initiative (ADNI)

*Denotes equal contribution

Correspondence: Paul M. Thompson, pthomp@usc.edu

Table of Contents

1. Supplementary Data 3
1.1 ADNI3 Excluded Participants
Supplementary Table 1 3
1.2 Available Cognitive Evaluations
Supplementary Table 2 3
1.3 Demographic Data for Cognitively Normal Controls
Supplementary Table 3 4
2. ADNI2 and ADNI3 dMRI Associations with Age in Cognitively Normal Controls 4
2.1 ADNI2 and ADNI3 *Pooled* dMRI ROI Associations with Age
Supplementary Table 4 4
2.2 dMRI ROI Associations with Age *by Protocol*
2.2.1 GE54 Associations with Age
Supplementary Table 5 5
2.2.2 P33 Associations with Age
Supplementary Table 6 6
2.2.3 P36 Associations with Age
Supplementary Table 7 6
2.2.4 S31 Associations with Age
Supplementary Table 8 7
2.2.5 S55 Associations with Age
Supplementary Table 9 7
2.2.6 S127 Associations with Age
Supplementary Table 10 8
2.2.7 ADNI2 Associations with Age
Supplementary Table 11 8
2.3 dMRI Associations with Age *by Protocol* in Age and Sex Matched Subsets of N=12
Supplementary Figure 1 9
3. ComBat Harmonization of ADNI2 and ADNI3 Protocols 10

3.1 Protocol Differences in dMRI Indices After ComBat
Supplementary Figure 2.. 10
3.2 ADNI2 and ADNI3 *Pooled* dMRI ROI Associations with Age After ComBat
3.2.1 Associations with Age After ComBat
Supplementary Table 12.. 11
3.2.2 dMRI Effect Sizes Before and After ComBat
Supplementary Figure 3.. 12
3.3 dMRI ROI Associations with Age After ComBat *by Protocol*
3.3.1 Full WM Associations with Age
Supplementary Figure 4.. 13
3.3.2 Fx/ST dMRI Associations with Age
Supplementary Figure 5.. 14
3.3.3 GCC dMRI Associations with Age
Supplementary Figure 6.. 15
4. ADNI3 dMRI Associations with Clinical Measures.. 16
4.1 Regional dMRI Measures: *Pooled* Associations with Cognitive Measures
4.1.1 CDR-sob Associations
Supplementary Table 13.. 16
4.1.2 ADAS-cog Associations
Supplementary Table 14.. 17
4.1.3 MMSE Associations
Supplementary Table 15.. 17
4.2. dMRI Associations with Clinical Measures *by Protocol*
4.2.1 Full WM Clinical Associations
Supplementary Figure 7.. 18
4.2.2 CGH Clinical Associations
Supplementary Figure 8.. 19
4.2.3 Fx/ST Clinical Associations
Supplementary Figure 9.. 20
4.2.4 FA^{DTI} vs FA^{TDF} Associations with CDR-sob
Supplementary Figure 10.. 21
4.3 Brain Maps of *Pooled* dMRI Associations with Cognitive Measures
4.3.1 ADAS-cog Associations
Supplementary Figure 11.. 22
4.3.2 MMSE Associations
Supplementary Figure 12.. 23
4.4 ROI Size vs CDR-sob Effect Size
Supplementary Figure 13.. 24
4.5 Regional dMRI Measures: *Pooled* Associations with Diagnosis
Supplementary Table 16.. 25
4.6 Brain Maps of dMRI *Pooled* Associations with Diagnosis
Supplementary Figure 14.. 26

1. Supplementary Data

1.1 ADNI3 Excluded Participants

Supplementary Table 1. Of the 381 raw dMRI scans available for download from the ADNI database as of April 2018 (https://ida.loni.usc.edu/), 64 were excluded from the study for the reasons summarized below. Participants with missing diagnosis, age, or with no available cognitive scores were excluded. Images were manually inspected for quality, and were removed if there were scanner-related artifacts or pathology inconsistent with the ADNI inclusion criteria, or if the data available from the IDA was corrupted. Protocols that included participants with overlapping exclusion criteria are marked with an asterisk.

Exclusion Criteria	GE36	GE54*	P33	P36*	P54	S31*	S55	S127*	TOTAL
No diagnosis available	2	3	-	1	-	2	5	4	17
No age available	-	-	-	-	-	1	-	-	1
All 3 cognitive scores unavailable	-	5	-	3	-	4	13	5	30
Artifacts/severe motion in DWI	-	-	-	-	-	2	2	-	3
Corrupt DWI file/missing DICOM files	1	-	3	-	-	-	-	-	4
Failed to complete protocol acquisition	-	-	-	1	-	-	-	1	2
No bval/bvec file available	-	-	-	-	-	2	1	-	3
Brain pathology	-	-	1	-	-	-	-	-	1
Total usable subjects n<10	9	-	-	-	-	-	-	-	9
Protocol not yet in effect	-	-	-	-	X	-	-	-	NA
TOTAL subjects excluded	12	6	4	3	X	10	20	9	64

*overlapping subject(s)

1.2 Available Cognitive Evaluations

Supplementary Table 2. Some ADNI3 participants only completed a subset of cognitive assessments. Here, we break down the number of participants that had available scores for each cognitive evaluation.

Protocol	MMSE	CDR-sob	ADAS-cog
GE54	64	65	61
P33	24	24	20
P36	19	19	18
S31	35	36	27
S55	153	152	132
S127	20	20	20
TOTAL	315	316	278

1.3 Demographic Data for Cognitively Normal Controls

Supplementary Table 3. Demographics for ADNI2 and ADNI3 cognitively normal (CN) participants, by protocol.

Protocol		All			Matched		
		Total N	Age Mean ± SD	Male N (%)	Total N	Age Mean ± SD	Male N (%)
ADNI3	GE54	45	75.8±7.0	20 (44.4)	12	76.4±3.8	4 (33.3)
	P33	17	78.5±7.3	9 (52.9)	12	77.0±6.3	4 (33.3)
	P36	12	75.9±6.4	4 (33.3)	12	75.9±6.4	4 (33.3)
	S31	21	71.6±7.0	8 (38.1)	12	75.3±6.9	4 (33.3)
	S55	96	73.5±7.6	36 (37.5)	12	75.6±3.5	4 (33.3)
	S127	16	75.2±6.0	6 (37.5)	12	76.5±4.8	4 (33.3)
	Total	207	74.5±7.4	83 (40.1)	72	76.1±5.3	24 (33.3)
ADNI2	GE46	59	72.4±6.6	24 (40.7)	12	76.5±3.8	4 (33.3)
Total		266	74.0±7.3	107 (40.2)	84	76.2±5.1	28 (33.3)

2. ADNI2 and ADNI3 dMRI Associations with Age in Cognitively Normal Controls

2.1 ADNI2 and ADNI3 *Pooled* dMRI ROI Associations with Age

Supplementary Table 4. *P*-values and corresponding effect sizes (*d*-values) are reported for associations between age and 5 dMRI anisotropy and diffusivity indices in pooled ADNI2 and ADNI3 CN participants (N = 266). For each test, the ROIs are ordered by *d*-value. Regions that were significant after FDR (q = 0.05) or Bonferroni (α = 0.05) multiple comparisons correction are delineated by a dotted or solid line respectively.

Age

FA^{DTI}			FA^{TDF}			AxD^{DTI}			MD^{DTI}			RD^{DTI}		
ROI	*p*-value	*d*-value	ROI	*p*-value	*d*-value	ROI	*p*-value	*d*-value	ROI	*p*-value	*d*-value	ROI	*p*-value	*d*-value
Fx/ST	5.09E-17	-1.27	Fx/ST	5.07E-21	-1.46	SS	2.76E-20	1.43	SS	3.74E-20	1.42	CGC	2.59E-19	1.38
ACR	5.29E-17	-1.27	Full WM	8.04E-20	-1.40	Full WM	8.13E-18	1.31	CGC	1.77E-18	1.34	ACR	3.26E-18	1.33
Full WM	1.42E-14	-1.15	CGC	2.30E-18	-1.33	GCC	8.49E-18	1.31	Full WM	4.96E-18	1.32	Full WM	1.01E-17	1.30
TAP	9.61E-13	-1.06	SS	8.09E-18	-1.31	EC	4.47E-17	1.27	IFO	1.04E-17	1.30	SS	1.39E-17	1.30
BCC	1.20E-12	-1.05	GCC	2.06E-17	-1.29	Fx/ST	3.00E-16	1.23	GCC	1.34E-17	1.30	GCC	7.20E-16	1.21
CC	1.18E-11	-1.00	CP	1.18E-16	-1.25	ALIC	4.06E-15	1.18	ACR	4.27E-17	1.27	CGH	7.21E-16	1.21
GCC	1.57E-11	-0.99	ACR	3.04E-16	-1.23	CGH	2.43E-14	1.14	CGH	2.46E-16	1.24	IFO	9.05E-16	1.21
CGC	4.39E-10	-0.91	BCC	8.66E-16	-1.21	RLIC	4.02E-14	1.13	ALIC	2.68E-16	1.24	Fx/ST	1.86E-15	1.19
SFO	2.31E-9	-0.87	CC	1.34E-15	-1.20	IFO	4.06E-14	1.13	EC	3.15E-16	1.23	SCR	2.43E-15	1.19
PTR	6.56E-9	-0.84	TAP	2.57E-15	-1.19	UNC	8.23E-14	1.11	Fx/ST	7.56E-16	1.21	ALIC	3.28E-15	1.18
ALIC	1.63E-8	-0.82	CGH	2.66E-14	-1.14	CC	1.11E-13	1.10	UNC	4.03E-15	1.18	EC	3.57E-15	1.18
CGH	3.85E-8	-0.79	EC	3.43E-14	-1.13	TAP	1.55E-13	1.10	RLIC	8.07E-15	1.16	UNC	1.03E-14	1.16
SCC	4.55E-8	-0.79	SCR	3.84E-14	-1.13	BCC	2.14E-13	1.09	CP	1.30E-14	1.15	RLIC	1.21E-13	1.10
SS	5.30E-7	-0.72	RLIC	1.04E-13	-1.11	CP	7.16E-13	1.06	SLF	2.76E-14	1.14	TAP	2.56E-13	1.09
EC	9.50E-7	-0.70	ALIC	1.07E-13	-1.11	SLF	1.54E-12	1.04	SCR	4.87E-14	1.12	CP	5.77E-13	1.07
CP	1.32E-5	-0.62	SFO	1.07E-13	-1.10	ACR	1.83E-12	1.04	TAP	1.48E-13	1.10	BCC	2.92E-12	1.03
IFO	6.22E-5	-0.57	PTR	6.22E-13	-1.07	PLIC	1.57E-11	0.99	BCC	7.72E-13	1.06	SLF	6.28E-12	1.01
RLIC	7.15E-5	-0.56	PLIC	1.68E-12	-1.04	SCR	1.94E-11	0.99	PLIC	1.09E-12	1.05	PTR	1.42E-11	0.99
SCR	3.06E-4	-0.51	SLF	1.16E-11	-1.00	SFO	3.69E-11	0.97	CC	3.64E-12	1.02	CC	2.80E-11	0.98
UNC	5.26E-4	-0.49	SCC	1.73E-11	-0.99	CGC	1.20E-10	0.94	PTR	1.40E-11	0.99	SFO	5.12E-11	0.96
SLF	7.35E-3	-0.38	IFO	2.86E-10	-0.92	PTR	2.89E-10	0.92	SFO	3.73E-11	0.97	PLIC	1.05E-9	0.89
PLIC	0.012	-0.35	UNC	5.49E-10	-0.90	SCC	6.14E-10	0.90	SCC	3.08E-8	0.80	SCC	1.57E-7	0.75
PCR	0.075	-0.25	PCR	5.36E-7	-0.72	PCR	2.15E-8	0.81	PCR	1.32E-7	0.76	PCR	8.40E-7	0.70
CST	0.202	-0.18	CST	9.30E-6	-0.63	CST	4.33E-8	0.79	CST	1.40E-6	0.69	CST	8.99E-6	0.63
Significant ROIs (N)			Significant ROIs (N)			Significant ROIs (N)			Significant ROIs (N)			Significant ROIs (N)		
FDR Threshold		22	FDR Threshold		24	FDR Threshold		24	FDR Threshold		24	FDR Threshold		24
Bonferroni Threshold		20	Bonferroni Threshold		24	Bonferroni Threshold		24	Bonferroni Threshold		24	Bonferroni Threshold		24

FDR Threshold — Bonferroni Threshold (*p*-value ≤ 0.05/24 = 0.0021) — Bonferroni & FDR Thresholds

2.2 dMRI ROI Associations with Age *by Protocol*

2.2.1 GE54 Associations with Age

Supplementary Table 5. For CN participants scanned with protocol GE54 (N = 45), *p*-values and corresponding effect sizes (*d*-values) are reported for associations between age and 5 dMRI anisotropy and diffusivity indices. For each test, the ROIs are ordered by *d*-value. Regions that were significant after FDR (q = 0.05) or Bonferroni (α = 0.05) multiple comparisons correction are delineated by a dotted or solid line respectively.

GE54 Age

FA^{DTI}		
ROI	*p*-value	*d*-value
ACR	1.02E-5	-1.74
ALIC	4.14E-5	-1.58
Fx/ST	1.01E-4	-1.48
Full WM	1.16E-4	-1.47
EC	4.53E-4	-1.31
SLF	9.41E-4	-1.22
SS	9.52E-4	-1.22
GCC	1.06E-3	-1.21
PTR	1.41E-3	-1.17
CC	2.98E-3	-1.08
SFO	3.49E-3	-1.06
TAP	3.78E-3	-1.05
CST	4.79E-3	-1.02
BCC	4.89E-3	-1.02
RLIC	5.61E-3	-1.00
SCC	7.67E-3	-0.96
CGH	8.34E-3	-0.95
UNC	0.010	-0.92
CGC	0.012	-0.89
CP	0.017	-0.85
PLIC	0.024	-0.80
IFO	0.044	-0.71
SCR	0.145	-0.50
PCR	0.622	0.17
Significant ROIs (N)		
FDR Threshold		22
Bonferroni Threshold		9

FA^{TDF}		
ROI	*p*-value	*d*-value
EC	1.54E-7	-2.21
ALIC	4.11E-7	-2.10
SLF	4.48E-6	-1.83
ACR	7.67E-6	-1.77
CGH	8.19E-6	-1.77
Full WM	8.26E-6	-1.76
CGC	1.84E-5	-1.68
SS	2.35E-5	-1.65
PLIC	2.86E-5	-1.63
CST	6.13E-5	-1.54
SCR	6.71E-5	-1.53
CP	8.44E-5	-1.50
RLIC	9.86E-5	-1.49
IFO	1.29E-4	-1.45
GCC	1.80E-4	-1.42
Fx/ST	2.63E-4	-1.37
SCC	2.63E-4	-1.37
CC	4.94E-4	-1.30
PCR	6.88E-4	-1.26
PTR	7.31E-4	-1.25
UNC	7.85E-4	-1.24
TAP	1.03E-3	-1.21
SFO	3.03E-3	-1.08
BCC	3.27E-3	-1.07
Significant ROIs (N)		
FDR Threshold		24
Bonferroni Threshold		22

AxD^{DTI}		
ROI	*p*-value	*d*-value
EC	3.81E-8	2.37
ALIC	4.67E-7	2.08
CGH	8.12E-7	2.02
CGC	8.20E-7	2.02
CST	6.22E-6	1.80
Full WM	1.21E-5	1.72
PLIC	2.30E-5	1.65
SCC	3.50E-5	1.60
PCR	3.51E-5	1.60
SS	5.14E-5	1.56
CP	5.63E-5	1.55
CC	1.90E-4	1.41
SCR	2.11E-4	1.40
RLIC	2.21E-4	1.39
SLF	2.37E-4	1.38
GCC	5.63E-4	1.28
TAP	5.84E-4	1.28
UNC	7.65E-4	1.25
ACR	8.72E-4	1.23
BCC	2.26E-3	1.11
Fx/ST	2.48E-3	1.10
PTR	2.92E-3	1.08
SFO	6.19E-3	0.98
IFO	0.051	0.68
Significant ROIs (N)		
FDR Threshold		23
Bonferroni Threshold		19

MD^{DTI}		
ROI	*p*-value	*d*-value
ALIC	2.20E-8	2.43
CGH	4.10E-8	2.36
EC	5.39E-8	2.33
PLIC	3.03E-7	2.13
SLF	6.20E-7	2.05
CGC	9.66E-7	2.00
RLIC	5.23E-6	1.82
Full WM	9.79E-6	1.75
CST	1.02E-5	1.74
CP	1.30E-5	1.71
SS	2.05E-5	1.66
PCR	2.16E-5	1.66
SCR	2.54E-5	1.64
ACR	5.07E-5	1.56
UNC	6.56E-5	1.53
SCC	1.81E-4	1.42
GCC	3.75E-4	1.33
CC	4.21E-4	1.32
TAP	5.99E-4	1.28
Fx/ST	1.35E-3	1.18
PTR	1.42E-3	1.17
IFO	1.83E-3	1.14
BCC	2.20E-3	1.12
SFO	5.40E-3	1.00
Significant ROIs (N)		
FDR Threshold		24
Bonferroni Threshold		23

RD^{DTI}		
ROI	*p*-value	*d*-value
ALIC	6.56E-8	2.31
CGH	8.73E-8	2.27
EC	1.45E-7	2.22
SLF	3.65E-7	2.11
PLIC	7.40E-7	2.03
RLIC	6.26E-6	1.80
SCR	9.02E-6	1.75
CGC	1.10E-5	1.73
Full WM	1.18E-5	1.72
ACR	1.91E-5	1.67
CST	2.13E-5	1.66
SS	2.43E-5	1.64
UNC	2.50E-5	1.64
CP	5.82E-5	1.55
PCR	1.11E-4	1.47
GCC	5.01E-4	1.30
SCC	5.37E-4	1.29
TAP	7.11E-4	1.25
CC	7.30E-4	1.25
IFO	8.49E-4	1.23
Fx/ST	1.07E-3	1.21
PTR	1.21E-3	1.19
BCC	2.59E-3	1.10
SFO	5.20E-3	1.01
Significant ROIs (N)		
FDR Threshold		24
Bonferroni Threshold		22

FDR Threshold — Bonferroni Threshold (*p*-value ≤ 0.05/24 = 0.0021) — Bonferroni & FDR Thresholds

2.2.2 P33 Associations with Age

Supplementary Table 6. For CN participants scanned with protocol P33 (N = 17), *p*-values and corresponding effect sizes (*d*-values) are reported for associations between age and 5 dMRI anisotropy and diffusivity indices. For each test, the ROIs are ordered by *d*-value. Regions that were significant after FDR (q = 0.05) or Bonferroni (α = 0.05) multiple comparisons correction are delineated by a dotted or solid line respectively.

P33

FA^{DTI}			FA^{TDF}			AxD^{DTI}			MD^{DTI}			RD^{DTI}		
ROI	*p*-value	*d*-value	ROI	*p*-value	*d*-value	ROI	*p*-value	*d*-value	ROI	*p*-value	*d*-value	ROI	*p*-value	*d*-value
CGC	0.02	-1.58	CGC	0.003	-2.27	IFO	0.001	2.93	CGC	0.005	2.14	CGC	0.003	2.33
Fx/ST	0.07	-1.22	CP	0.01	-1.96	CP	0.001	2.53	CP	0.01	1.99	CP	0.02	1.67
ACR	0.12	-1.03	IFO	0.05	-1.35	CGC	0.02	1.62	IFO	0.01	1.96	IFO	0.04	1.39
GCC	0.21	-0.79	Fx/ST	0.05	-1.30	GCC	0.03	1.49	GCC	0.06	1.26	GCC	0.09	1.13
CP	0.22	-0.79	GCC	0.06	-1.25	SFO	0.04	1.40	SFO	0.08	1.15	ACR	0.10	1.09
BCC	0.29	-0.67	CGH	0.10	-1.10	CGH	0.06	1.28	ACR	0.11	1.06	SFO	0.12	1.02
SS	0.38	-0.55	ACR	0.10	-1.08	BCC	0.08	1.14	CGH	0.13	0.98	Fx/ST	0.16	0.90
CC	0.45	-0.47	SFO	0.12	-1.03	PLIC	0.10	1.07	SCR	0.14	0.95	SCR	0.18	0.86
Full WM	0.47	-0.45	SCR	0.12	-1.01	SCR	0.12	1.01	Fx/ST	0.16	0.92	CGH	0.22	0.79
PCR	0.49	-0.43	Full WM	0.19	-0.85	Full WM	0.14	0.96	SLF	0.17	0.88	BCC	0.23	0.76
SCR	0.66	-0.27	BCC	0.19	-0.85	Fx/ST	0.15	0.94	BCC	0.21	0.81	SLF	0.27	0.70
CST	0.78	-0.18	EC	0.24	-0.75	ACR	0.15	0.93	Full WM	0.23	0.77	Full WM	0.28	0.69
CGH	0.80	-0.15	SS	0.29	-0.67	UNC	0.20	0.83	EC	0.31	0.64	CC	0.37	0.57
SCC	0.87	-0.10	SLF	0.32	-0.62	SLF	0.21	0.80	CC	0.35	0.59	EC	0.40	0.53
SFO	0.90	-0.08	CC	0.35	-0.59	EC	0.21	0.80	SS	0.38	0.55	SS	0.43	0.49
SLF	0.92	-0.06	CST	0.36	-0.57	CC	0.25	0.74	CST	0.47	0.46	CST	0.51	0.41
IFO	0.99	0.01	PLIC	0.61	-0.32	ALIC	0.31	0.64	PLIC	0.50	0.42	PCR	0.76	0.19
EC	0.85	0.11	ALIC	0.61	-0.31	SS	0.32	0.63	UNC	0.59	0.33	ALIC	0.85	0.12
TAP	0.76	0.19	RLIC	0.77	-0.18	RLIC	0.37	0.57	ALIC	0.64	0.29	UNC	0.89	0.09
ALIC	0.75	0.19	SCC	0.77	-0.18	CST	0.39	0.54	RLIC	0.79	0.16	SCC	0.90	0.08
PTR	0.72	0.22	PCR	0.81	-0.15	PTR	0.66	0.27	PCR	0.80	0.16	PLIC	0.98	0.02
RLIC	0.27	0.70	UNC	0.85	-0.12	PCR	0.90	0.07	SCC	0.88	0.09	PTR	1.00	0.0004
PLIC	0.22	0.78	PTR	0.92	-0.06	SCC	0.99	0.01	PTR	0.90	0.08	RLIC	0.88	-0.09
UNC	0.04	1.37	TAP	0.81	0.15	TAP	0.81	-0.15	TAP	0.81	-0.15	TAP	0.80	-0.15
Significant ROIs (N)			Significant ROIs (N)			Significant ROIs (N)			Significant ROIs (N)			Significant ROIs (N)		
FDR Threshold		0	FDR Threshold		0	FDR Threshold		2	FDR Threshold		0	FDR Threshold		0
Bonferroni Threshold:		0	Bonferroni Threshold:		0	Bonferroni Threshold:		2	Bonferroni Threshold:		0	Bonferroni Threshold:		0

FDR Threshold — Bonferroni Threshold (*p*-value ≤ 0.05/24 = 0.0021) — Bonferroni & FDR Thresholds

2.2.3 P36 Associations with Age

Supplementary Table 7. For CN participants scanned with protocol P36 (N = 12), *p*-values and corresponding effect sizes (*d*-values) are reported for associations between age and 5 dMRI anisotropy and diffusivity indices. For each test, the ROIs are ordered by *d*-value. No associations were significant after correction for multiple comparisons.

P36

FA^{DTI}			FA^{TDF}			AxD^{DTI}			MD^{DTI}			RD^{DTI}		
ROI	*p*-value	*d*-value	ROI	*p*-value	*d*-value	ROI	*p*-value	*d*-value	ROI	*p*-value	*d*-value	ROI	*p*-value	*d*-value
Fx/ST	0.02	-2.86	BCC	0.02	-3.00	BCC	0.03	2.72	BCC	0.03	2.79	BCC	0.03	2.73
IFO	0.08	-1.94	Fx/ST	0.04	-2.48	GCC	0.03	2.57	GCC	0.04	2.50	GCC	0.06	2.23
BCC	0.13	-1.61	GCC	0.06	-2.14	CC	0.08	1.98	CC	0.09	1.90	Fx/ST	0.07	2.06
TAP	0.17	-1.44	CC	0.13	-1.61	Fx/ST	0.10	1.80	Fx/ST	0.09	1.86	CC	0.11	1.77
ACR	0.20	-1.32	SFO	0.14	-1.56	SFO	0.12	1.70	CGC	0.10	1.83	CGC	0.11	1.73
SS	0.22	-1.25	ALIC	0.26	-1.14	CGC	0.14	1.57	SFO	0.13	1.60	SFO	0.15	1.51
CP	0.23	-1.21	CGC	0.29	-1.07	ALIC	0.23	1.23	UNC	0.16	1.49	ACR	0.15	1.50
EC	0.28	-1.09	IFO	0.30	-1.03	UNC	0.23	1.22	ACR	0.18	1.38	UNC	0.22	1.26
PTR	0.35	-0.93	EC	0.31	-1.01	ACR	0.30	1.05	EC	0.32	0.99	EC	0.31	1.01
GCC	0.35	-0.93	ACR	0.38	-0.86	EC	0.35	0.93	ALIC	0.36	0.89	IFO	0.33	0.97
Full WM	0.55	-0.58	Full WM	0.44	-0.75	Full WM	0.36	0.90	IFO	0.36	0.89	Full WM	0.35	0.92
CC	0.68	-0.39	TAP	0.44	-0.75	SLF	0.39	0.85	Full WM	0.41	0.80	SS	0.37	0.89
RLIC	0.69	-0.38	SS	0.45	-0.73	SCC	0.46	0.71	TAP	0.43	0.77	TAP	0.38	0.86
SFO	0.76	-0.29	SLF	0.53	-0.60	IFO	0.48	0.68	SS	0.51	0.63	ALIC	0.48	0.69
CGH	0.81	-0.22	RLIC	0.56	-0.56	PLIC	0.49	0.67	RLIC	0.54	0.58	RLIC	0.53	0.60
CGC	1.00	0.002	PLIC	0.58	-0.52	TAP	0.50	0.65	SLF	0.57	0.54	PTR	0.57	0.55
UNC	0.96	0.04	UNC	0.75	-0.31	SCR	0.51	0.64	SCR	0.60	0.50	SCR	0.67	0.40
ALIC	0.93	0.09	SCC	0.80	-0.24	RLIC	0.56	0.55	SCC	0.64	0.45	SLF	0.69	0.38
SCR	0.92	0.09	SCR	0.87	-0.15	SS	0.57	0.54	PLIC	0.76	0.29	SCC	0.85	0.17
SLF	0.74	0.32	PTR	0.90	-0.12	PCR	0.68	0.40	PTR	0.79	0.25	PCR	1.00	0.01
PCR	0.58	0.53	PCR	0.98	-0.02	PTR	0.80	-0.24	PCR	0.87	0.15	PLIC	0.95	-0.06
SCC	0.48	0.69	CGH	0.91	0.11	CGH	0.68	-0.39	CGH	0.74	-0.32	CP	0.81	-0.23
PLIC	0.43	0.77	CP	0.88	0.14	CST	0.57	-0.54	CP	0.60	-0.50	CGH	0.80	-0.24
CST	0.36	0.90	CST	0.53	0.60	CP	0.39	-0.84	CST	0.52	-0.63	CST	0.50	-0.65
Significant ROIs (N)			Significant ROIs (N)			Significant ROIs (N)			Significant ROIs (N)			Significant ROIs (N)		
FDR Threshold		0	FDR Threshold		0	FDR Threshold		0	FDR Threshold		0	FDR Threshold		0
Bonferroni Threshold:		0	Bonferroni Threshold:		0	Bonferroni Threshold:		0	Bonferroni Threshold:		0	Bonferroni Threshold:		0

FDR Threshold — Bonferroni Threshold (*p*-value ≤ 0.05/24 = 0.0021) — Bonferroni & FDR Thresholds

2.2.4 S31 Associations with Age

Supplementary Table 8. For CN participants scanned with protocol S31 (N = 21), *p*-values and corresponding effect sizes (*d*-values) are reported for associations between age and 5 dMRI anisotropy and diffusivity indices. For each test, the ROIs are ordered by *d*-value. No associations were significant after correction for multiple comparisons.

	FA^{DTI}			FA^{TDF}			AxD^{DTI}			MD^{DTI}			RD^{DTI}		
	ROI	*p*-value	*d*-value	ROI	*p*-value	*d*-value	ROI	*p*-value	*d*-value	ROI	*p*-value	*d*-value	ROI	*p*-value	*d*-value
S31	SFO	0.01	-1.80	BCC	0.04	-1.34	SS	0.06	1.20	SS	0.06	1.20	BCC	0.06	1.22
	BCC	0.04	-1.36	Fx/ST	0.06	-1.20	PCR	0.14	0.92	BCC	0.07	1.15	SS	0.07	1.14
	ACR	0.13	-0.94	PLIC	0.08	-1.10	TAP	0.14	0.92	SFO	0.17	0.85	SFO	0.10	1.01
	EC	0.20	-0.79	CP	0.09	-1.08	BCC	0.14	0.90	SLF	0.17	0.84	SLF	0.11	1.00
	IFO	0.21	-0.77	TAP	0.12	-0.96	Fx/ST	0.16	0.85	UNC	0.17	0.83	UNC	0.15	0.89
	Fx/ST	0.21	-0.77	SCR	0.14	-0.92	SCR	0.18	0.83	SCR	0.18	0.82	CP	0.16	0.86
	CC	0.22	-0.75	SS	0.17	-0.85	EC	0.18	0.82	Fx/ST	0.18	0.81	SCR	0.19	0.81
	GCC	0.23	-0.74	SFO	0.19	-0.81	SLF	0.29	0.64	PCR	0.20	0.78	Fx	0.20	0.78
	Full WM	0.24	-0.72	CC	0.21	-0.76	GCC	0.32	0.59	TAP	0.20	0.78	GCC	0.24	0.72
	SLF	0.25	-0.70	GCC	0.25	-0.70	UNC	0.34	0.57	CP	0.20	0.78	EC	0.24	0.72
	CGC	0.29	-0.64	Full WM	0.31	-0.62	SFO	0.35	0.57	EC	0.21	0.76	TAP	0.24	0.71
	UNC	0.29	-0.64	UNC	0.31	-0.61	CC	0.36	0.55	GCC	0.24	0.71	CC	0.26	0.68
	SS	0.35	-0.56	RLIC	0.37	-0.54	CGC	0.42	0.49	CC	0.28	0.65	CGC	0.30	0.63
	SCR	0.39	-0.52	CGC	0.42	-0.48	RLIC	0.45	0.45	CGC	0.32	0.60	PCR	0.31	0.61
	PTR	0.41	-0.49	SLF	0.43	-0.47	Full WM	0.46	0.44	Full WM	0.38	0.53	Full WM	0.31	0.61
	TAP	0.42	-0.49	EC	0.43	-0.47	SCC	0.73	0.20	RLIC	0.54	0.36	IFO	0.36	0.55
	CP	0.59	-0.32	CST	0.50	0.40	CP	0.74	0.20	ACR	0.61	0.30	ACR	0.39	0.52
	SCC	0.64	-0.28	PCR	0.52	-0.38	ALIC	0.77	0.17	IFO	0.62	0.29	RLIC	0.56	0.35
	CGH	0.66	-0.26	ACR	0.55	-0.35	PLIC	0.81	0.14	SCC	0.68	0.24	ALIC	0.66	0.26
	ALIC	0.82	-0.13	SCC	0.57	-0.33	CGH	0.86	0.11	CGH	0.74	0.20	SCC	0.67	0.26
	RLIC	0.88	0.09	ALIC	0.68	-0.24	IFO	0.89	0.08	ALIC	0.75	0.19	PTR	0.67	0.26
	PCR	0.62	0.30	IFO	0.75	-0.19	PTR	0.98	-0.01	PTR	0.77	0.17	CGH	0.70	0.23
	PLIC	0.50	0.40	CGH	0.82	-0.13	ACR	0.90	-0.07	PLIC	0.99	0.01	PLIC	0.86	-0.10
	CST	0.04	1.34	PTR	0.84	-0.12	CST	0.05	-1.24	CST	0.07	-1.13	CST	0.09	-1.06
	Significant ROIs (N)			Significant ROIs (N)			Significant ROIs (N)			Significant ROIs (N)			Significant ROIs (N)		
	FDR Threshold		0	FDR Threshold		0	FDR Threshold		0	FDR Threshold		0	FDR Threshold		0
	Bonferroni Threshold		0	Bonferroni Threshold:		0	Bonferroni Threshold		0	Bonferroni Threshold		0	Bonferroni Threshold		0

········ FDR Threshold —— Bonferroni Threshold (*p*-value ≤ 0.05/24 = 0.0021) ═══ Bonferroni & FDR Thresholds

2.2.5 S55 Associations with Age

Supplementary Table 9. For CN participants scanned with protocol S55 (N = 96), *p*-values and corresponding effect sizes (*d*-values) are reported for associations between age and 5 dMRI anisotropy and diffusivity indices. For each test, the ROIs are ordered by *d*-value. Regions that were significant after FDR (q = 0.05) or Bonferroni (α = 0.05) multiple comparisons correction are delineated by a dotted or solid line respectively.

	FA^{DTI}			FA^{TDF}			AxD^{DTI}			MD^{DTI}			RD^{DTI}		
	ROI	*p*-value	*d*-value	ROI	*p*-value	*d*-value	ROI	*p*-value	*d*-value	ROI	*p*-value	*d*-value	ROI	*p*-value	*d*-value
S55 Age	ACR	6.20E-8	-1.37	Full WM	1.86E-10	-1.67	IFO	2.18E-10	1.67	CGC	2.71E-13	2.01	CGC	1.01E-11	1.82
	Fx/ST	1.19E-7	-1.33	CGC	5.67E-10	-1.62	Full WM	1.22E-9	1.58	SS	2.87E-11	1.77	ACR	5.41E-11	1.74
	Full WM	1.55E-7	-1.32	CP	1.09E-9	-1.58	SS	3.32E-9	1.52	ACR	1.05E-10	1.70	FullWM	1.17E-10	1.70
	CC	2.64E-7	-1.29	SS	2.04E-9	-1.55	GCC	9.8E-9	1.47	Full WM	1.29E-10	1.69	SS	2.80E-10	1.65
	BCC	2.86E-7	-1.28	Fx/ST	3.27E-9	-1.52	UNC	1.40E-8	1.45	CP	6.16E-10	1.61	CGH	2.00E-9	1.55
	GCC	1.03E-6	-1.21	ACR	9.33E-9	-1.47	CGH	1.67E-8	1.44	CGH	8.12E-10	1.60	UNC	2.92E-9	1.53
	TAP	1.13E-6	-1.21	SFO	1.48E-8	-1.44	ACR	2.06E-8	1.43	UNC	1.40E-9	1.57	CP	7.45E-9	1.48
	SFO	2.69E-5	-1.02	GCC	1.60E-8	-1.44	CP	2.31E-8	1.42	IFO	2.04E-9	1.55	SCR	1.21E-8	1.45
	SCC	4.06E-5	-0.99	CC	2.27E-8	-1.42	CC	8.15E-8	1.35	GCC	4.00E-9	1.51	GCC	1.32E-8	1.45
	CGC	5.80E-5	-0.97	RLIC	3.18E-8	-1.40	EC	8.26E-8	1.35	SLF	7.84E-9	1.48	RLIC	1.02E-7	1.34
	CGH	6.19E-5	-0.97	BCC	8.59E-8	-1.35	CST	8.33E-8	1.35	PLIC	8.59E-9	1.47	CC	1.28E-7	1.33
	PTR	2.04E-4	-0.89	CGH	2.22E-7	-1.30	SLF	1.57E-7	1.32	ALIC	3.71E-8	1.39	BCC	1.34E-7	1.32
	ALIC	5.30E-4	-0.82	EC	4.19E-7	-1.26	ALIC	1.66E-7	1.31	RLIC	5.15E-8	1.38	IFO	1.42E-7	1.32
	SS	1.60E-3	-0.75	SCR	5.69E-7	-1.24	CGC	3.05E-7	1.28	CC	7.49E-8	1.36	ALIC	2.11E-7	1.30
	RLIC	1.79E-3	-0.74	TAP	6.31E-7	-1.24	BCC	3.61E-7	1.27	SCR	1.20E-7	1.33	EC	3.66E-7	1.27
	EC	3.14E-3	-0.70	UNC	7.17E-7	-1.23	SFO	4.83E-7	1.25	BCC	1.37E-7	1.32	SLF	7.72E-7	1.23
	CP	3.84E-3	-0.68	PTR	8.68E-7	-1.22	RLIC	6.03E-7	1.24	EC	1.48E-7	1.32	SFO	9.83E-7	1.21
	UNC	0.013	-0.58	SCC	9.03E-7	-1.22	Fx/ST	6.25E-7	1.24	SFO	6.74E-7	1.23	PTR	1.53E-6	1.19
	IFO	0.026	-0.52	PLIC	1.68E-6	-1.18	PLIC	7.55E-7	1.23	Fx/ST	9.67E-7	1.21	Fx/ST	1.62E-6	1.18
	SCR	0.122	-0.36	SLF	3.06E-6	-1.15	PTR	2.51E-6	1.16	PTR	9.91E-7	1.21	TAP	2.00E-6	1.17
	PCR	0.149	-0.33	ALIC	1.14E-5	-1.07	TAP	3.30E-6	1.14	TAP	1.93E-6	1.17	PCR	2.77E-6	1.15
	PLIC	0.169	-0.32	PCR	6.12E-5	-0.97	SCC	3.32E-6	1.14	PCR	2.49E-6	1.16	PLIC	3.61E-6	1.14
	SLF	0.303	-0.24	IFO	1.91E-4	-0.89	SCR	1.09E-5	1.07	SCC	7.07E-6	1.10	SCC	1.99E-5	1.04
	CST	0.782	-0.06	CST	1.05E-3	-0.78	PCR	2.44E-5	1.02	CST	9.56E-6	1.08	CST	1.27E-4	0.92
	Significant ROIs (N)			Significant ROIs (N)			Significant ROIs (N)			Significant ROIs (N)			Significant ROIs (N)		
	FDR Threshold		19	FDR Threshold		24	FDR Threshold		24	FDR Threshold		24	FDR Threshold		24
	Bonferroni Threshold		15	Bonferroni Threshold		24	Bonferroni Threshold		24	Bonferroni Threshold		24	Bonferroni Threshold		24

········ FDR Threshold —— Bonferroni Threshold (*p*-value ≤ 0.05/24 = 0.0021) ═══ Bonferroni & FDR Thresholds

2.2.6 S127 Associations with Age

Supplementary Table 10. For CN participants scanned with protocol S127 (N = 16), *p*-values and corresponding effect sizes (*d*-values) are reported for associations between age and 5 dMRI anisotropy and diffusivity indices. For each test, the ROIs are ordered by *d*-value. Regions that were significant after FDR (q = 0.05) or Bonferroni (α = 0.05) multiple comparisons correction are delineated by a dotted or solid line respectively.

S127 Age

FA^{DTI}			FA^{TDF}			AxD^{DTI}			MD^{DTI}			RD^{DTI}		
ROI	*p*-value	*d*-value	ROI	*p*-value	*d*-value	ROI	*p*-value	*d*-value	ROI	*p*-value	*d*-value	ROI	*p*-value	*d*-value
ACR	0.004	-2.84	ALIC	0.009	-2.42	CGH	0.001	3.96	CGH	0.001	3.44	CGH	0.002	3.14
EC	0.005	-2.68	TAP	0.015	-2.18	GCC	0.007	2.55	ACR	0.004	2.79	ACR	0.002	3.08
CGH	0.006	-2.66	GCC	0.018	-2.09	TAP	0.010	2.37	GCC	0.007	2.57	RLIC	0.003	3.02
Full WM	0.008	-2.49	CGH	0.018	-2.09	SS	0.010	2.37	UNC	0.008	2.5	EC	0.004	2.82
IFO	0.010	-2.39	CC	0.021	-2.03	ALIC	0.016	2.15	RLIC	0.011	2.31	IFO	0.005	2.72
CGC	0.024	-1.96	Full WM	0.022	-2.00	ACR	0.022	2.00	TAP	0.016	2.17	UNC	0.007	2.57
TAP	0.026	-1.93	SCC	0.026	-1.93	Full WM	0.023	1.98	ALIC	0.015	2.17	GCC	0.01	2.37
RLIC	0.033	-1.81	PLIC	0.028	-1.90	PCR	0.026	1.92	SS	0.016	2.14	ALIC	0.018	2.1
PTR	0.036	-1.78	SFO	0.041	-1.72	Fx/ST	0.027	1.91	EC	0.019	2.08	Full WM	0.019	2.07
CC	0.047	-1.66	SS	0.048	-1.65	PTR	0.035	1.80	Full WM	0.021	2.04	TAP	0.019	2.06
SCC	0.052	-1.61	Fx/ST	0.049	-1.64	CC	0.039	1.74	PCR	0.021	2.03	PTR	0.022	2.01
ALIC	0.053	-1.61	PTR	0.049	-1.64	SFO	0.041	1.72	PTR	0.024	1.97	PCR	0.026	1.93
GCC	0.053	-1.61	BCC	0.051	-1.63	SCC	0.052	1.61	IFO	0.024	1.97	SS	0.03	1.87
BCC	0.069	-1.49	SLF	0.059	-1.56	CST	0.054	1.60	Fx/ST	0.041	1.72	PLIC	0.029	1.87
Fx/ST	0.074	-1.46	ACR	0.065	-1.51	SCR	0.068	1.49	PLIC	0.044	1.69	Fx/ST	0.047	1.66
UNC	0.074	-1.45	CP	0.066	-1.51	BCC	0.081	1.41	CC	0.054	1.59	SCR	0.053	1.61
SLF	0.097	-1.33	PCR	0.088	-1.37	PLIC	0.117	1.24	SCC	0.058	1.57	CC	0.058	1.56
PLIC	0.105	-1.29	RLIC	0.095	-1.34	UNC	0.126	1.21	SCR	0.058	1.56	SCC	0.061	1.54
SFO	0.315	-0.76	SCR	0.100	-1.31	RLIC	0.138	1.16	SFO	0.06	1.55	CST	0.069	1.48
CST	0.331	-0.73	IFO	0.106	-1.29	EC	0.160	1.09	CST	0.062	1.54	SFO	0.073	1.46
SS	0.577	-0.41	UNC	0.107	-1.28	CP	0.163	1.09	BCC	0.097	1.33	CGC	0.082	1.41
PCR	0.757	-0.23	CST	0.119	-1.24	CGC	0.292	0.80	CGC	0.132	1.19	BCC	0.098	1.32
SCR	0.767	-0.22	EC	0.139	-1.16	IFO	0.377	0.66	CP	0.172	1.06	SLF	0.122	1.22
CP	0.966	0.03	CGC	0.157	-1.11	SLF	0.463	0.55	SLF	0.207	0.97	CP	0.193	1.00
Significant ROIs (N)			Significant ROIs (N)			Significant ROIs (N)			Significant ROIs (N)			Significant ROIs (N)		
FDR Threshold		5	FDR Threshold		0	FDR Threshold		1	FDR Threshold		13	FDR Threshold		11
Bonferroni Threshold		0	Bonferroni Threshold		0	Bonferroni Threshold		1	Bonferroni Threshold		1	Bonferroni Threshold		2

········ FDR Threshold — Bonferroni Threshold (*p*-value ≤ 0.05/24 = 0.0021) ═ Bonferroni & FDR Thresholds

2.2.7 ADNI2 Associations with Age

Supplementary Table 11. For CN participants scanned with the ADNI2 protocol (N = 59), *p*-values and corresponding effect sizes (*d*-values) are reported for associations between age and 5 dMRI anisotropy and diffusivity indices. For each test, the ROIs are ordered by *d*-value. Regions that were significant after FDR (q = 0.05) or Bonferroni (α = 0.05) multiple comparisons correction are delineated by a dotted or solid line respectively.

ADNI2 Age

FA^{DTI}			FA^{TDF}			AxD^{DTI}			MD^{DTI}			RD^{DTI}		
ROI	*p*-value	*d*-value	ROI	*p*-value	*d*-value	ROI	*p*-value	*d*-value	ROI	*p*-value	*d*-value	ROI	*p*-value	*d*-value
Fx/ST	1.01E-5	-1.55	Fx/ST	3.07E-6	-1.66	Fx/ST	2.13E-5	1.48	Fx/ST	1.44E-5	1.51	Fx/ST	1.31E-5	1.52
Full WM	8.91E-4	-1.10	CGH	4.94E-5	-1.40	SS	4.28E-5	1.41	RLIC	1.23E-4	1.31	CGH	2.78E-4	1.22
TAP	1.26E-3	-1.07	Full WM	7.93E-5	-1.35	RLIC	2.17E-4	1.25	SS	1.50E-4	1.29	SS	3.93E-4	1.19
ALIC	1.32E-3	-1.06	CGC	8.16E-5	-1.35	SLF	3.49E-4	1.20	CGH	2.28E-4	1.24	ALIC	4.58E-4	1.17
GCC	2.89E-3	-0.98	SS	1.33E-4	-1.30	CGH	3.94E-4	1.19	Full WM	5.24E-4	1.16	RLIC	5.02E-4	1.16
PTR	3.27E-3	-0.96	RLIC	1.46E-4	-1.29	Full WM	4.65E-4	1.17	ALIC	5.35E-4	1.16	Full WM	6.36E-4	1.14
CC	6.40E-3	-0.89	EC	2.56E-4	-1.23	GCC	4.79E-4	1.17	GCC	5.37E-4	1.16	CGC	6.86E-4	1.13
SCC	6.53E-3	-0.88	GCC	2.94E-4	-1.22	EC	1.31E-3	1.06	IFO	7.32E-4	1.12	IFO	9.84E-4	1.09
ACR	9.82E-3	-0.83	PTR	2.96E-4	-1.22	PTR	1.50E-3	1.05	CGC	9.22E-4	1.10	GCC	1.02E-3	1.09
SCR	0.012	-0.81	TAP	7.82E-4	-1.12	TAP	1.73E-3	1.03	PTR	1.10E-3	1.08	PTR	1.08E-3	1.08
BCC	0.013	-0.80	BCC	8.66E-4	-1.11	CC	2.07E-3	1.01	TAP	1.43E-3	1.05	TAP	1.48E-3	1.05
CGC	0.020	-0.75	ACR	1.01E-3	-1.09	BCC	2.14E-3	1.01	SLF	1.59E-3	1.04	SCR	1.71E-3	1.03
PCR	0.020	-0.74	CC	1.08E-3	-1.08	IFO	3.07E-3	0.97	EC	1.88E-3	1.02	EC	2.98E-3	0.97
CGH	0.024	-0.73	IFO	1.09E-3	-1.08	ALIC	4.47E-3	0.93	SCR	3.37E-3	0.96	ACR	4.22E-3	0.93
SFO	0.039	-0.66	ALIC	1.31E-3	-1.06	SCC	6.02E-3	0.89	ACR	4.44E-3	0.93	CC	6.79E-3	0.88
SS	0.047	-0.63	SCC	2.41E-3	-1.00	CGC	6.46E-3	0.88	CC	4.57E-3	0.92	BCC	7.31E-3	0.87
IFO	0.059	-0.60	SCR	2.43E-3	-1.00	CST	9.77E-3	0.84	BCC	4.67E-3	0.92	SLF	8.45E-3	0.85
CST	0.060	-0.60	SLF	4.11E-3	-0.94	ACR	0.012	0.82	SCC	0.011	0.82	SCC	0.015	0.79
CP	0.069	-0.58	CST	4.40E-3	-0.93	SCR	0.012	0.81	CST	0.015	0.79	CP	0.019	0.76
RLIC	0.084	-0.55	CP	4.54E-3	-0.93	UNC	0.012	0.81	CP	0.016	0.77	CST	0.019	0.75
EC	0.167	-0.43	PLIC	0.018	-0.76	CP	0.028	0.70	PLIC	0.017	0.77	UNC	0.037	0.66
PLIC	0.183	-0.42	UNC	0.033	-0.68	PLIC	0.033	0.68	UNC	0.024	0.72	PLIC	0.044	0.64
SLF	0.318	-0.31	PCR	0.043	-0.65	PCR	0.062	0.59	PCR	0.050	0.62	PCR	0.047	0.63
UNC	0.860	0.05	SFO	0.060	-0.60	SFO	0.145	0.46	SFO	0.105	0.51	SFO	0.090	0.53
Significant ROIs (N)			Significant ROIs (N)			Significant ROIs (N)			Significant ROIs (N)			Significant ROIs (N)		
FDR Threshold		14	FDR Threshold		23	FDR Threshold		22	FDR Threshold		22	FDR Threshold		23
Bonferroni Threshold:		9	Bonferroni Threshold:		15	Bonferroni Threshold:		11	Bonferroni Threshold:		13	Bonferroni Threshold:		12

········ FDR Threshold — Bonferroni Threshold (*p*-value ≤ 0.05/24 = 0.0021) ═ Bonferroni & FDR Thresholds

2.3 dMRI Associations with Age *by Protocol* in Age and Sex Matched Subsets of N=12

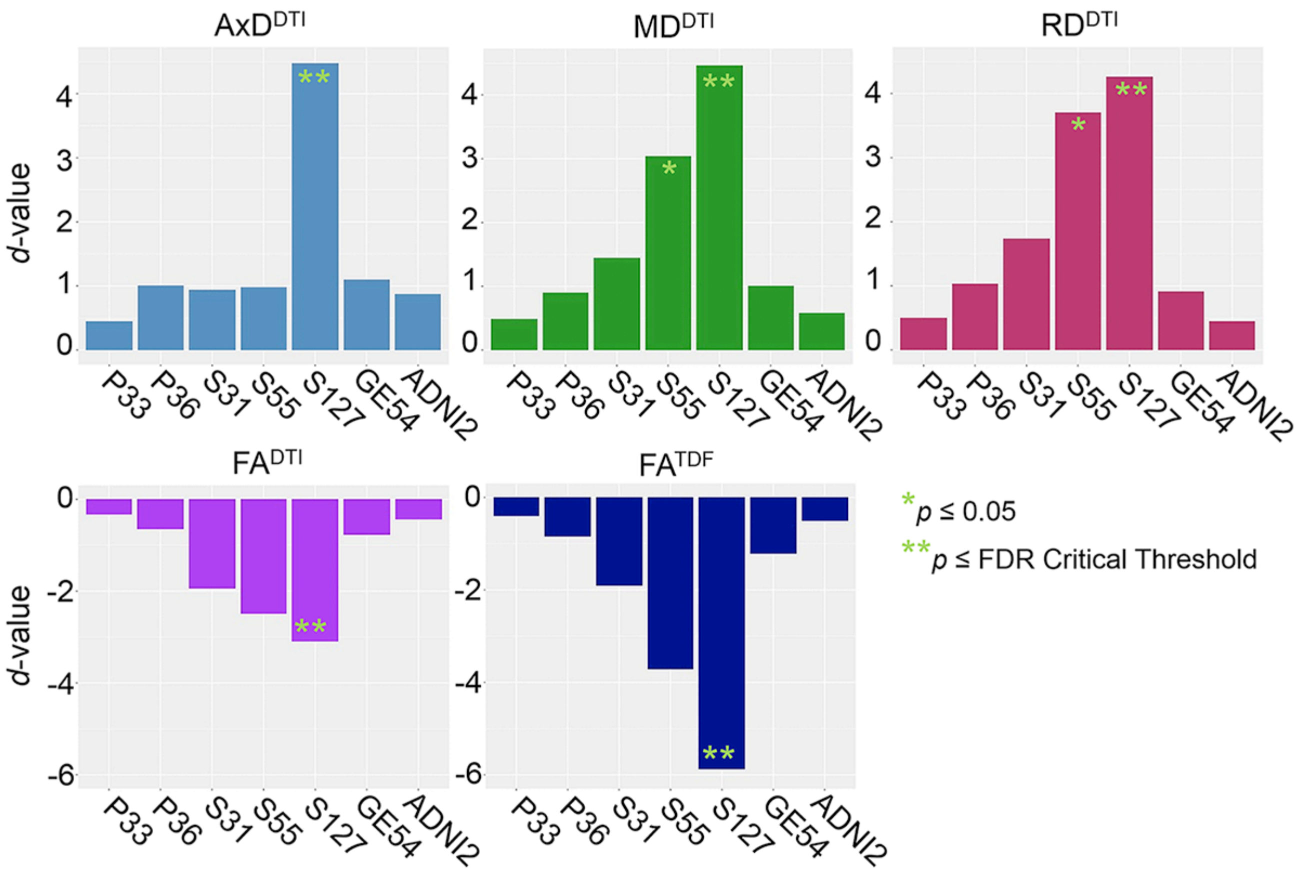

Supplementary Figure 1. Effect sizes (*d*-values) from 12 CN sex- and age-matched controls from each ADNI protocol (**Supplementary Table 3**) show the direction of dMRI associations with age in the full WM were consistent across protocols. While direct comparisons of effect sizes were underpowered, findings suggested larger effect sizes for protocol S127, the protocol with greatest total number of diffusion-weighted (b = 1000 s/mm^2) and non-diffusion sensitized (b_0) gradients, followed by S55, the protocol with the second greatest number of diffusion-weighted and b_0 gradients.

3. ComBat Harmonization of ADNI2 and ADNI3 Protocols

3.1 Protocol Differences in dMRI Indices After ComBat

(A)

	ANCOVA				
	FA^{DTI}	AxD^{DTI}	MD^{DTI}	RD^{DTI}	FA^{TDF}
CST	0.40*	0.43*	0.41*	0.40*	0.41*
CP	0.07	0.14	0.12	0.1	0.1
ALIC	0.11	0.12	0.14	0.14	0.12
PLIC	0.1	0.1	0.12	0.11	0.11
RLIC	0.1	0.12	0.12	0.12	0.12
PTR	0.06	0.13	0.14	0.13	0.13
ACR	0.15	0.14	0.15	0.15	0.13
SCR	0.05	0.09	0.1	0.09	0.1
PCR	0.07	0.09	0.08	0.07	0.09
CGC	0.06	0.11	0.12	0.13	0.13
CGH	0.1	0.12	0.11	0.11	0.12
Fx/ST	0.1	0.15	0.15	0.14	0.16
SLF	0.11	0.1	0.1	0.1	0.1
SFO	0.08	0.11	0.11	0.11	0.12
IFO	0.08	0.15	0.13	0.12	0.14
SS	0.07	0.17	0.16	0.13	0.13
EC	0.1	0.17	0.15	0.15	0.15
UNC	0.06	0.07	0.08	0.08	0.08
GCC	0.13	0.14	0.15	0.14	0.16
BCC	0.12	0.09	0.1	0.12	0.14
SCC	0.08	0.09	0.1	0.1	0.12
CC	0.12	0.1	0.12	0.12	0.14
TAP	0.13	0.14	0.14	0.15	0.16
Full WM	0.1	0.13	0.13	0.12	0.13
Significant ROIs	1	1	1	1	1
FDR Threshold	3.81E-8	2.17E-9	1.21E-8	4.01E-8	9.12E-9

* $p \leq$ FDR Critical Threshold

d-value: 0.65, 0.3, 0

(B)

	Pairwise Tests					
	FA^{DTI}	AxD^{DTI}	MD^{DTI}	RD^{DTI}	FA^{TDF}	Protocol Total
ADNI2	3	4	4	4	3	18 (2.50%)
GE54	2	2	2	2	2	10 (1.39%)
P33	1	0	0	0	0	1 (0.001%)
P36	1	4	4	3	2	14 (1.94%)
S31	6	2	2	2	0	12 (1.67%)
S55	2	2	2	2	2	10 (1.39%)
S127	1	2	2	1	1	7 (0.83%)
Index Total	16 (3.17%)	16 (3.17%)	16 (3.17%)	14 (2.78%)	10 (1.98%)	

Significant Tests (N): 6, 3, 0

Supplementary Figure 2. (A) *d*-values from the ANCOVAs assessing differences in dMRI indices between protocols, for each of the 24 ROIs, after ComBat harmonization; across dMRI indices, the CST was the only region where significant protocol differences remained. **(B)** We report the number of times each protocol and each dMRI index showed significant differences in pairwise tests between protocols after ComBat harmonization (out of 504 tests per index and 720 tests per protocol). After ComBat, the number of pairwise tests for which each protocol showed significant differences in dMRI indices decreased by 93.8%.

3.2 ADNI2 and ADNI3 *Pooled* dMRI ROI Associations with Age After ComBat

3.2.1 Associations with Age After ComBat

Supplementary Table 12. *P*-values and corresponding effect sizes (*d*-values) are reported for ROI associations between age and 5 dMRI anisotropy and diffusivity indices in ADNI2 and ADNI3 protocols pooled after harmonization with ComBat. For each test, the ROIs are ordered by *d*-value. Regions that were significant after FDR ($q = 0.05$) or Bonferroni ($\alpha = 0.05$) multiple comparisons correction are delineated by a dotted or solid line respectively.

Age Post-ComBat

FA^{DTI}

ROI	*p*-value	*d*-value
Fx/ST	3.45E-17	-1.27
ACR	6.87E-17	-1.26
Full WM	3.89E-14	-1.12
BCC	1.60E-12	-1.04
GCC	4.45E-12	-1.02
CC	1.53E-11	-0.99
TAP	4.61E-11	-0.96
CGC	3.87E-10	-0.91
ALIC	5.42E-9	-0.84
SFO	5.44E-9	-0.84
PTR	8.68E-9	-0.83
CGH	3.12E-8	-0.80
SCC	8.65E-8	-0.77
EC	1.08E-6	-0.70
SS	1.62E-6	-0.68
CP	4.75E-5	-0.58
RLIC	5.48E-5	-0.57
IFO	1.01E-4	-0.55
SCR	9.21E-4	-0.47
UNC	1.63E-3	-0.44
SLF	0.013	-0.35
PLIC	0.019	-0.33
CST	0.104	-0.23
PCR	0.130	-0.21

Significant ROIs (N)	
FDR Threshold	22
Bonferroni Threshold	20

FA^{TDF}

ROI	*p*-value	*d*-value
Fx/ST	2.76E-20	-1.42
CGC	5.52E-20	-1.40
Full WM	9.96E-20	-1.39
SS	1.23E-17	-1.29
GCC	3.43E-17	-1.27
CP	1.38E-16	-1.24
ACR	2.40E-16	-1.23
CGH	5.44E-16	-1.21
BCC	2.73E-15	-1.18
CC	3.52E-15	-1.17
EC	4.78E-15	-1.17
SCR	1.99E-14	-1.14
RLIC	2.79E-14	-1.13
ALIC	1.90E-13	-1.09
TAP	4.37E-13	-1.07
SFO	1.57E-12	-1.04
PLIC	4.59E-12	-1.02
SLF	6.57E-12	-1.01
PTR	1.06E-11	-1.00
SCC	1.54E-11	-0.99
IFO	6.68E-11	-0.95
UNC	3.07E-10	-0.91
PCR	1.28E-7	-0.76
CST	2.71E-5	-0.59

Significant ROIs (N)	
FDR Threshold	24
Bonferroni Threshold	24

AxD^{DTI}

ROI	*p*-value	*d*-value
SS	5.33E-19	1.36
Full WM	1.26E-17	1.29
EC	2.60E-17	1.28
GCC	4.96E-17	1.26
CGH	1.19E-16	1.25
Fx/ST	1.23E-15	1.20
ALIC	2.52E-15	1.18
IFO	7.22E-15	1.16
CC	2.48E-14	1.13
UNC	2.71E-14	1.13
CGC	5.00E-14	1.12
CP	7.18E-14	1.11
RLIC	9.67E-14	1.10
BCC	1.16E-13	1.10
SLF	7.42E-13	1.06
ACR	3.04E-12	1.02
TAP	6.38E-12	1.01
PLIC	5.06E-11	0.96
SCC	6.72E-11	0.95
SCR	1.00E-10	0.94
SFO	2.98E-10	0.92
PTR	5.25E-10	0.90
PCR	8.92E-9	0.83
CST	1.05E-7	0.76

Significant ROIs (N)	
FDR Threshold	24
Bonferroni Threshold	24

MD^{DTI}

ROI	*p*-value	*d*-value
CGC	7.70E-22	1.49
SS	3.85E-20	1.41
CGH	7.22E-19	1.35
Full WM	1.21E-18	1.34
IFO	1.32E-18	1.34
GCC	2.07E-17	1.28
ALIC	3.84E-17	1.27
ACR	4.81E-17	1.26
EC	6.60E-17	1.26
CP	1.71E-15	1.19
Fx/ST	1.77E-15	1.19
SLF	1.79E-15	1.19
UNC	2.24E-15	1.18
RLIC	2.52E-15	1.18
SCR	3.83E-14	1.12
BCC	1.14E-13	1.10
PLIC	1.78E-13	1.09
CC	2.43E-13	1.08
TAP	6.02E-12	1.01
PTR	3.19E-11	0.97
SFO	3.32E-10	0.91
SCC	1.37E-9	0.88
PCR	1.58E-9	0.87
CST	1.91E-6	0.68

Significant ROIs (N)	
FDR Threshold	24
Bonferroni Threshold	24

RD^{DTI}

ROI	*p*-value	*d*-value
CGC	2.12E-21	1.47
Full WM	1.50E-18	1.34
ACR	2.38E-18	1.33
SS	3.22E-18	1.32
CGH	4.19E-18	1.32
IFO	2.62E-16	1.23
ALIC	3.05E-16	1.23
GCC	3.96E-16	1.22
SCR	6.65E-16	1.21
EC	7.29E-16	1.21
Fx/ST	2.50E-15	1.18
UNC	1.04E-14	1.15
RLIC	3.78E-14	1.12
CP	3.12E-13	1.08
BCC	3.41E-13	1.07
SLF	8.54E-13	1.05
CC	1.47E-12	1.04
TAP	9.04E-12	1.00
PTR	2.47E-11	0.98
PLIC	2.37E-10	0.92
SFO	4.68E-10	0.90
PCR	5.00E-9	0.84
SCC	9.83E-9	0.83
CST	9.93E-6	0.63

Significant ROIs (N)	
FDR Threshold	24
Bonferroni Threshold	24

FDR Threshold — Bonferroni Threshold (*p*-value ≤ 0.05/24 = 0.0021) — Bonferroni & FDR Thresholds

3.2.2 dMRI Effect Sizes Before and After ComBat

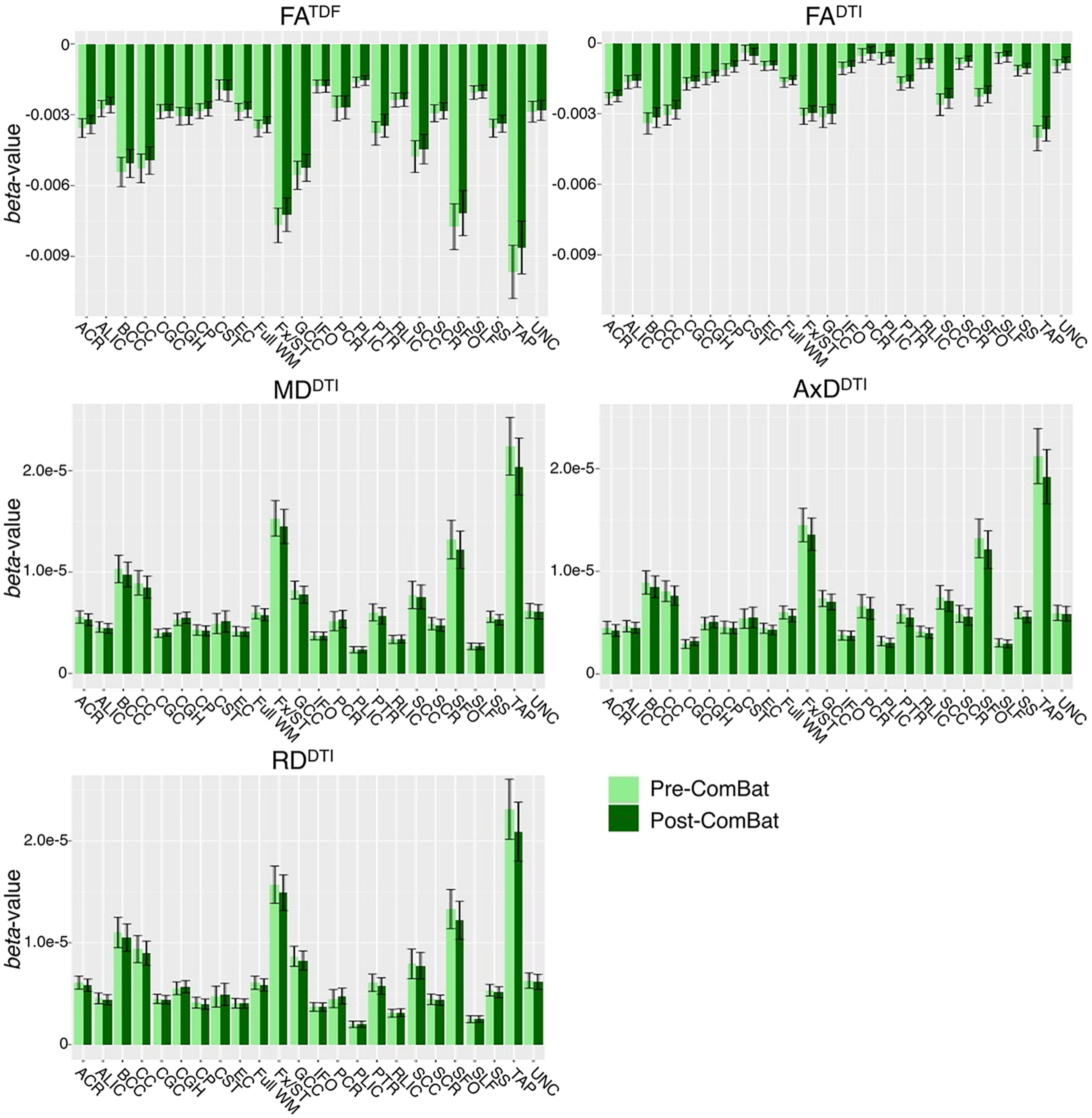

Supplementary Figure 3. Effect sizes (*beta*-values with error bars that represent the standard error) are plotted for the association between each diffusion index and age in CN controls from ADNI2 and ADNI3 protocols pooled together before and after ComBat harmonization. Compared to pre-ComBat analyses, effect sizes were marginally different across indices, but still within the standard error bounds. All associations were significant (FDR $q = 0.05$) except for FA^{DTI} in the CST and PCR.

3.3 dMRI ROI Associations with Age After ComBat *by Protocol*

3.3.1 Full WM Associations with Age

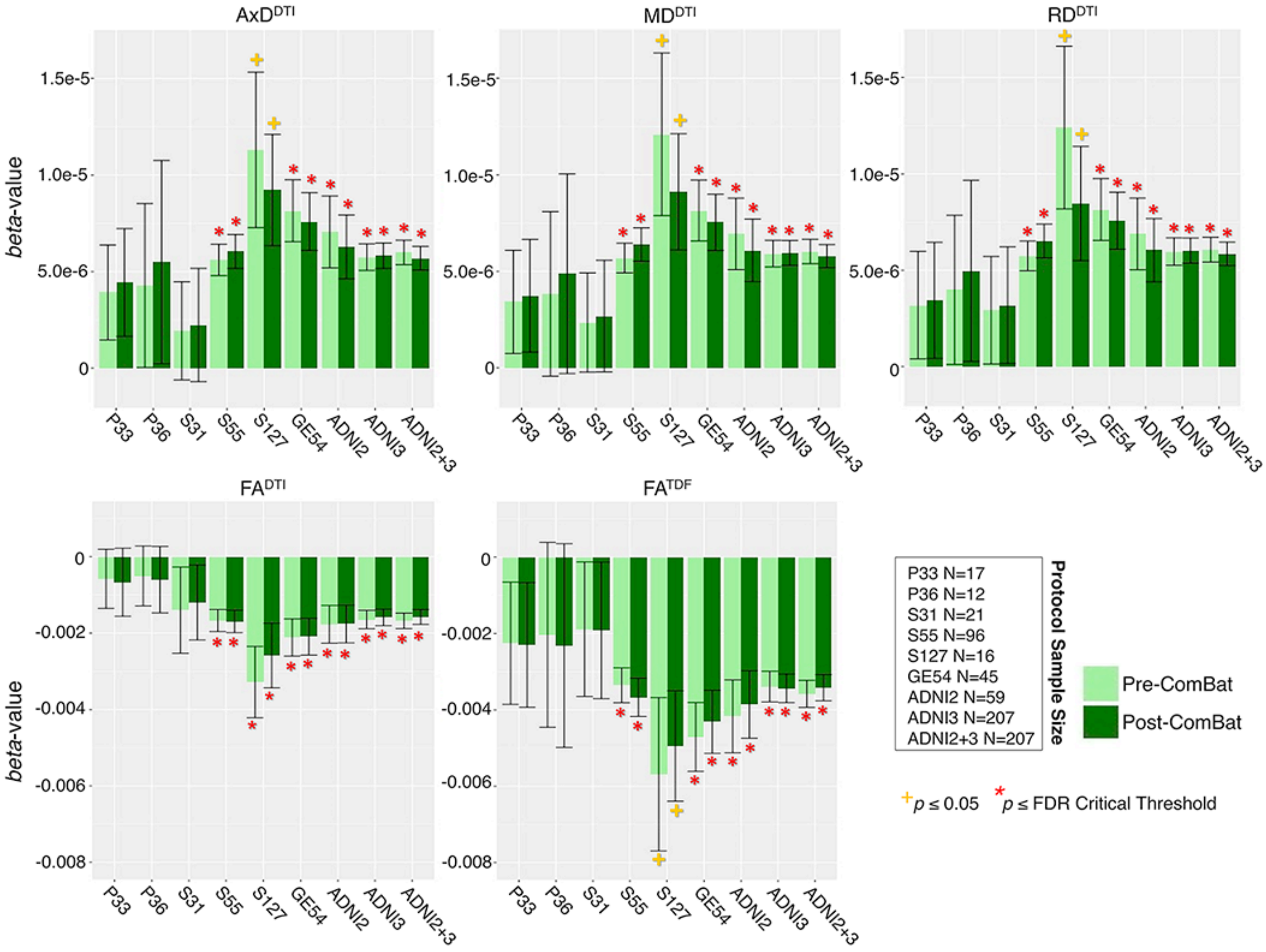

Supplementary Figure 4. For each protocol, the *beta*-values (error bars represent the standard error) are plotted for the association between each diffusion index in the full WM and age in CN controls, before and after ComBat harmonization. Compared to pre-ComBat analyses, effect sizes were marginally different across indices, but still within the standard error bounds.

3.3.2 Fx/ST dMRI Associations with Age

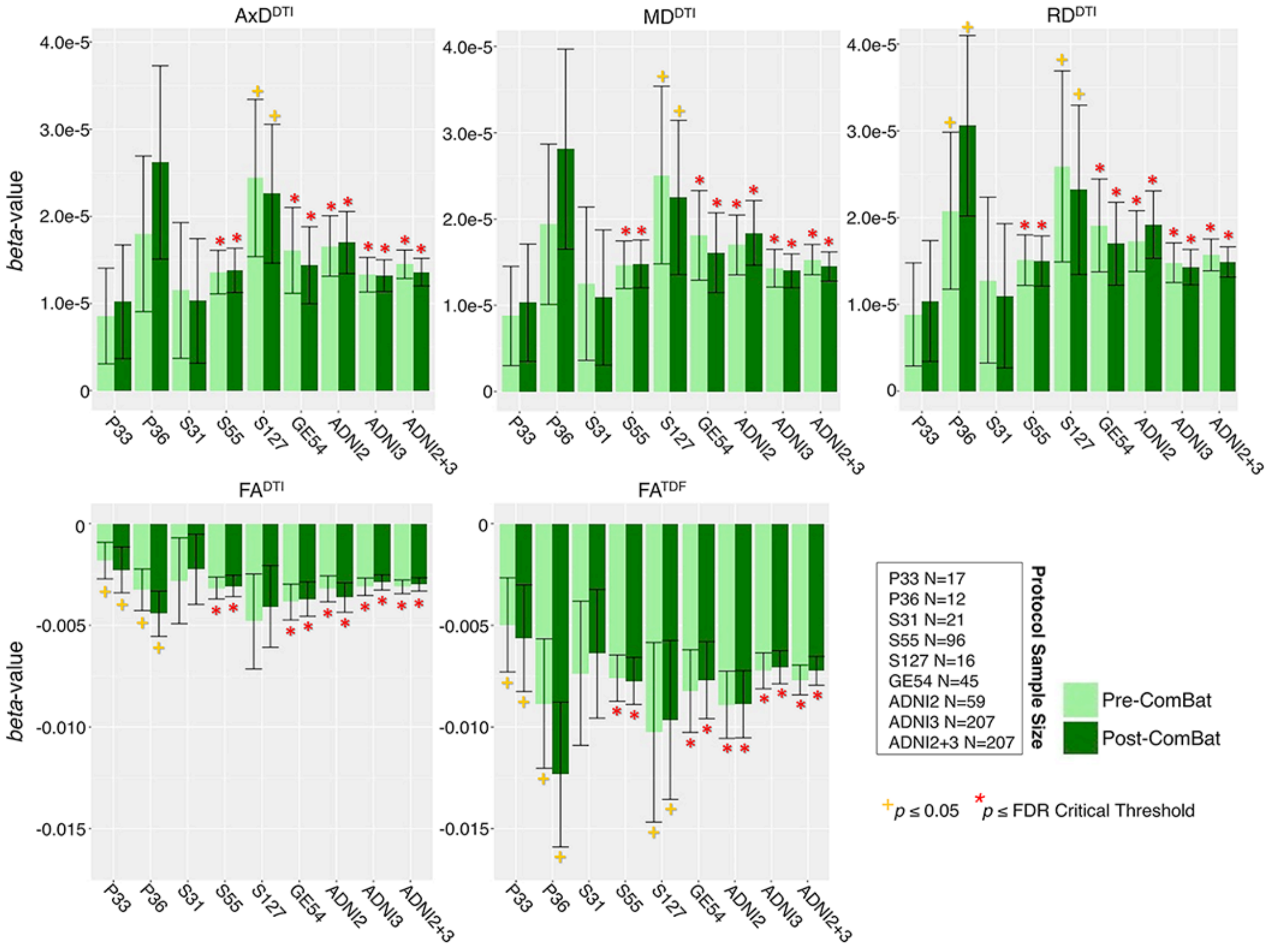

Supplementary Figure 5. For each protocol, the *beta*-values (error bars represent the standard error) are plotted for the association between each diffusion index in the fornix *(crus)* / *stria terminalis* (Fx/ST) and age in CN controls, before and after ComBat harmonization. Compared to pre-ComBat analyses, effect sizes were marginally different across indices, but still within the standard error bounds.

3.3.3 GCC dMRI Associations with Age

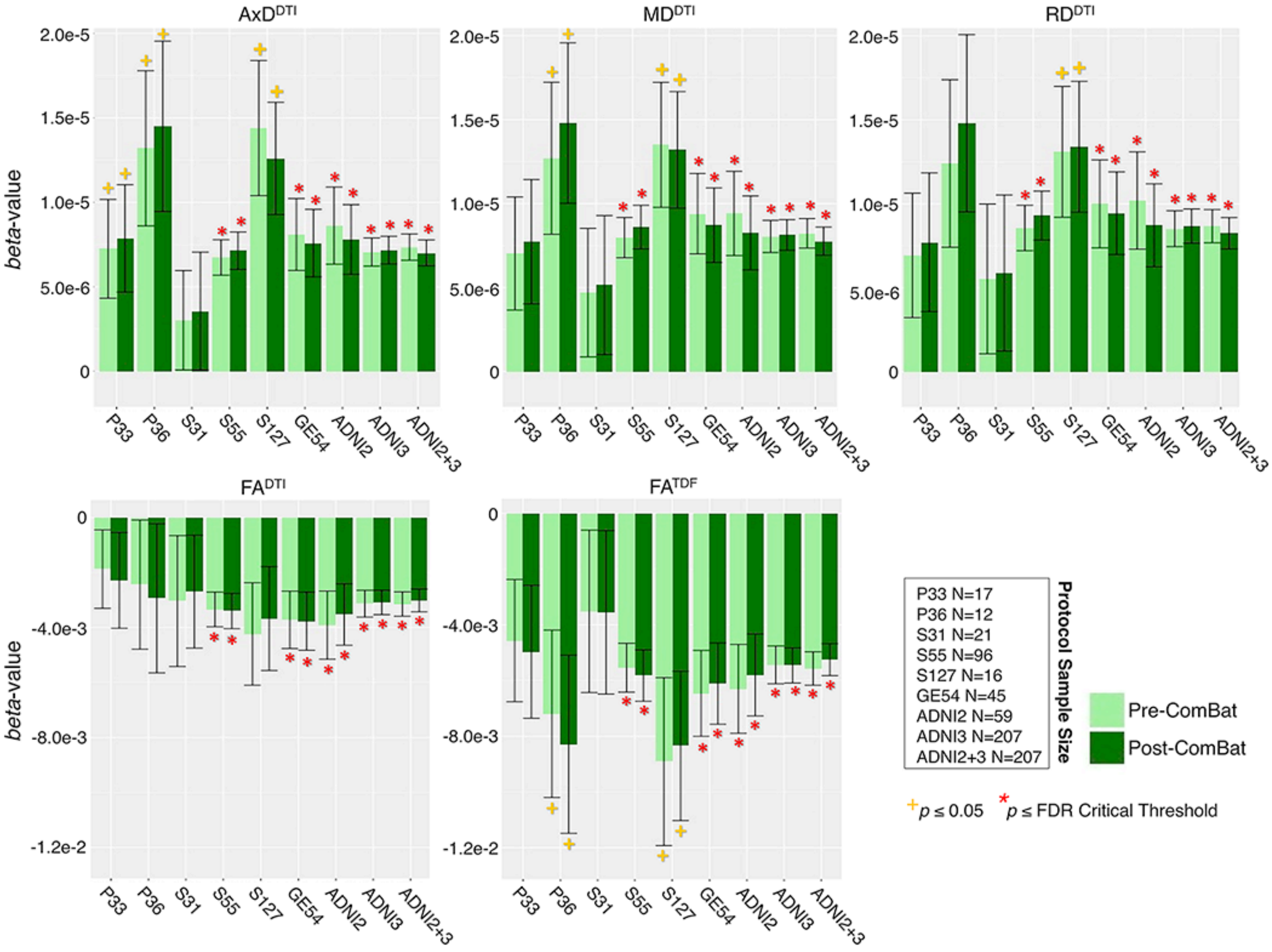

Supplementary Figure 6. For each protocol, the *beta*-values (error bars represent the standard error) are plotted for the association between each diffusion index in the corpus callosum genu (GCC) and age in CN controls, before and after ComBat harmonization. Compared to pre-ComBat analyses, effect sizes were only marginally different across indices and protocols, but still within the standard error bounds.

4. ADNI3 dMRI Associations with Clinical Measures

4.1 Regional dMRI Measures: *Pooled* Associations with Cognitive Measures

4.1.1 CDR-sob Associations

Supplementary Table 13. *P*-values and corresponding effect sizes *(d*-values) are reported for ROI associations between CDR-sob and dMRI indices across all pooled ADNI3 participants (N = 316). For each test, the ROIs are ordered by *d*-value. Regions that were significant after FDR ($q = 0.05$) or Bonferroni ($\alpha = 0.05$) multiple comparisons correction are delineated by a dotted or solid line respectively.

CDR-sob

FA^{DTI}

ROI	*p*-value	*d*-value
Fx/ST	7.01E-8	-0.68
GCC	1.06E-6	-0.61
CGH	1.26E-6	-0.61
BCC	1.94E-6	-0.60
IFO	2.13E-6	-0.60
CC	2.16E-6	-0.60
Full WM	9.52E-6	-0.55
PTR	1.36E-5	-0.54
TAP	1.68E-5	-0.54
CGC	3.00E-5	-0.52
SS	7.04E-5	-0.50
SCC	7.74E-5	-0.49
UNC	1.50E-4	-0.47
SFO	1.08E-3	-0.41
ACR	1.51E-3	-0.39
CP	1.97E-3	-0.38
ALIC	7.24E-3	-0.33
EC	0.019	-0.29
CST	0.025	-0.28
RLIC	0.042	-0.25
SLF	0.089	-0.21
PCR	0.622	0.06
SCR	0.649	0.06
PLIC	0.830	-0.03
Significant ROIs (N)		
FDR Threshold	19	
Bonferroni Threshold	16	

FA^{TDF}

ROI	*p*-value	*d*-value
UNC	1.39E-20	-1.24
CGH	8.86E-20	-1.21
SS	1.77E-12	-0.91
IFO	4.10E-12	-0.89
Fx/ST	1.55E-10	-0.82
CGC	8.95E-10	-0.78
GCC	1.20E-9	-0.77
CC	1.87E-9	-0.76
Full WM	2.13E-9	-0.76
BCC	1.08E-8	-0.73
SCC	7.42E-8	-0.68
PTR	2.29E-7	-0.65
CP	3.48E-6	-0.58
RLIC	8.81E-6	-0.56
SFO	1.07E-5	-0.55
SLF	1.73E-5	-0.54
TAP	5.02E-5	-0.51
ACR	6.57E-5	-0.50
EC	2.73E-4	-0.45
SCR	2.77E-4	-0.45
ALIC	2.84E-3	-0.37
PCR	7.73E-3	-0.33
PLIC	0.049	-0.24
CST	0.075	-0.22
Significant ROIs (N)		
FDR Threshold	22	
Bonferroni Threshold	20	

AxD^{DTI}

ROI	*p*-value	*d*-value
UNC	3.13E-23	1.34
CGH	4.59E-23	1.34
SS	6.15E-14	0.97
IFO	4.71E-11	0.84
GCC	1.20E-10	0.82
Fx/ST	3.89E-10	0.80
Full WM	5.88E-10	0.79
CC	1.26E-9	0.77
CGC	4.38E-9	0.75
BCC	1.08E-8	0.73
SCC	1.46E-7	0.66
RLIC	9.33E-7	0.62
ACR	1.72E-6	0.60
PTR	7.58E-6	0.56
SFO	1.23E-5	0.55
EC	1.28E-5	0.55
SLF	1.35E-5	0.54
TAP	2.55E-5	0.53
ALIC	4.07E-5	0.51
SCR	8.65E-5	0.49
CP	1.24E-4	0.48
PLIC	1.38E-4	0.48
PCR	1.78E-4	0.47
CST	0.035	0.12
Significant ROIs (N)		
FDR Threshold	23	
Bonferroni Threshold	23	

MD^{DTI}

ROI	p-value	d-value
UNC	3.47E-23	1.34
CGH	8.50E-22	1.29
IFO	3.34E-15	1.03
SS	9.36E-15	1.01
CGC	6.66E-14	0.97
GCC	2.51E-11	0.86
Full WM	2.62E-10	0.81
Fx/ST	5.82E-10	0.79
CC	2.23E-8	0.71
BCC	5.22E-8	0.69
RLIC	9.30E-8	0.67
ACR	3.76E-7	0.64
PTR	1.01E-6	0.62
SLF	2.23E-6	0.59
SCC	2.36E-6	0.59
CP	2.69E-6	0.59
ALIC	6.55E-6	0.57
TAP	6.82E-6	0.56
SFO	8.31E-6	0.56
EC	1.03E-5	0.55
SCR	9.54E-5	0.49
PCR	3.53E-4	0.44
PLIC	3.64E-4	0.44
CST	0.205	0.16
Significant ROIs (N)		
FDR Threshold	23	
Bonferroni Threshold	23	

RD^{DTI}

ROI	p-value	d-value
UNC	3.31E-22	1.31
CGH	4.17E-20	1.23
IFO	1.44E-14	1.00
SS	3.75E-14	0.98
CGC	2.91E-13	0.94
GCC	1.13E-10	0.83
Full WM	3.94E-10	0.80
Fx/ST	8.19E-10	0.78
CC	1.18E-7	0.67
BCC	1.63E-7	0.66
RLIC	2.60E-7	0.65
ACR	4.10E-7	0.64
PTR	6.98E-7	0.62
CP	3.59E-6	0.58
TAP	4.61E-6	0.57
SFO	7.82E-6	0.56
SCC	9.34E-6	0.56
ALIC	1.06E-5	0.55
EC	1.57E-5	0.54
SLF	2.02E-5	0.53
SCR	2.28E-4	0.46
PCR	1.61E-3	0.39
PLIC	6.92E-3	0.33
CST	0.167	0.17
Significant ROIs (N)		
FDR Threshold	23	
Bonferroni Threshold	22	

FDR Threshold (dotted line); Bonferroni Threshold (p-value $\leq 0.05/24 = 0.0021$) (solid line); Bonferroni & FDR Thresholds (double line)

4.1.2 ADAS-cog Associations

Supplementary Table 14. *P*-values and corresponding effect sizes (*d*-values) are reported for ROI associations between ADAS-cog diagnosis and dMRI indices across all ADNI3 participants pooled together (N = 278). For each test, the ROIs are ordered by *d*-value. Regions that were significant after FDR (q = 0.05) or Bonferroni (α = 0.05) multiple comparisons correction are delineated by a dotted or solid line respectively.

ADAS-cog

FA^{DTI}			FA^{TDF}			AxD^{DTI}			MD^{DTI}			RD^{DTI}		
ROI	*p*-value	*d*-value	ROI	*p*-value	*d*-value	ROI	*p*-value	*d*-value	ROI	*p*-value	*d*-value	ROI	*p*-value	*d*-value
Fx/ST	8.00E-7	-0.67	UNC	4.90E-13	-1.02	UNC	1.09E-13	1.05	UNC	1.71E-13	1.04	UNC	7.09E-13	1.01
CGH	4.52E-6	-0.62	CGH	2.70E-11	-0.93	CGH	4.00E-11	0.92	CGH	2.27E-11	0.93	CGH	5.35E-11	0.91
BCC	2.65E-5	-0.57	SS	1.18E-8	-0.78	SS	9.43E-11	0.90	SS	7.96E-11	0.90	SS	4.76E-10	0.86
CC	2.82E-5	-0.57	Fx/ST	5.57E-8	-0.74	Full WM	7.40E-9	0.80	Full WM	1.03E-8	0.79	Full WM	2.43E-8	0.77
TAP	6.32E-5	-0.54	Full WM	8.27E-8	-0.73	IFO	1.19E-8	0.78	IFO	1.46E-8	0.78	Fx/ST	9.26E-8	0.73
SCC	1.27E-4	-0.52	CC	1.60E-7	-0.72	PTR	3.15E-8	0.76	PTR	8.64E-8	0.73	PTR	2.78E-7	0.70
GCC	3.09E-4	-0.49	BCC	2.65E-7	-0.70	CC	6.13E-8	0.74	Fx/ST	8.94E-8	0.73	IFO	3.32E-7	0.70
Full WM	3.58E-4	-0.48	PTR	2.72E-7	-0.70	BCC	9.62E-8	0.73	RLIC	1.50E-7	0.72	RLIC	9.76E-7	0.67
PTR	1.12E-3	-0.44	SCC	3.46E-7	-0.70	Fx/ST	1.13E-7	0.73	CGC	6.94E-7	0.68	CGC	1.37E-6	0.66
SS	1.97E-3	-0.41	RLIC	6.35E-6	-0.61	SCC	2.55E-7	0.70	CC	7.77E-7	0.67	BCC	3.12E-6	0.63
CGC	3.80E-3	-0.39	IFO	7.76E-6	-0.61	RLIC	7.16E-7	0.68	BCC	9.04E-7	0.67	CC	3.25E-6	0.63
UNC	4.64E-3	-0.38	GCC	1.07E-5	-0.60	SLF	1.05E-5	0.60	SLF	2.69E-6	0.64	TAP	1.33E-5	0.59
ACR	8.12E-3	-0.35	CP	4.11E-5	-0.55	PCR	1.17E-5	0.59	SCC	3.54E-6	0.63	SCC	1.47E-5	0.59
IFO	9.55E-3	-0.35	SLF	5.54E-5	-0.54	GCC	4.35E-5	0.55	GCC	1.72E-5	0.58	SLF	2.62E-5	0.57
ALIC	0.022	-0.31	CGC	6.69E-5	-0.54	SCR	4.47E-5	0.55	TAP	2.01E-5	0.58	GCC	3.03E-5	0.56
EC	0.022	-0.31	SCR	1.94E-4	-0.50	TAP	7.77E-5	0.53	SCR	2.98E-5	0.56	ACR	5.58E-5	0.54
CP	0.026	-0.30	PLIC	3.50E-4	-0.48	ACR	8.36E-5	0.53	ACR	4.07E-5	0.55	SCR	6.33E-5	0.54
SFO	0.043	-0.27	TAP	4.58E-4	-0.47	CGC	8.87E-5	0.53	PCR	4.49E-5	0.55	ALIC	4.13E-4	0.47
SLF	0.091	-0.22	EC	5.18E-4	-0.47	ALIC	1.13E-4	0.52	PLIC	5.44E-5	0.54	PCR	4.48E-4	0.47
RLIC	0.142	-0.20	ACR	8.22E-4	-0.45	PLIC	1.95E-4	0.50	ALIC	1.38E-4	0.51	EC	4.95E-4	0.47
PLIC	0.211	-0.17	SFO	8.85E-4	-0.45	EC	3.56E-4	0.48	EC	3.47E-4	0.48	PLIC	5.04E-4	0.47
CST	0.246	-0.15	PCR	1.12E-3	-0.44	CP	1.55E-3	0.42	CP	3.86E-4	0.48	CP	7.45E-4	0.45
SCR	0.7501	0.04	ALIC	0.015	-0.32	SFO	1.85E-3	0.42	SFO	2.53E-3	0.40	SFO	3.11E-3	0.40
PCR	0.554	0.08	CST	0.071	-0.24	CST	0.09	0.22	CST	0.113	0.21	CST	0.132	0.20
Significant ROIs (N)			Significant ROIs (N)			Significant ROIs (N)			Significant ROIs (N)			Significant ROIs (N)		
FDR Threshold		17	FDR Threshold		23	FDR Threshold		23	FDR Threshold		23	FDR Threshold		23
Bonferroni Threshold		10	Bonferroni Threshold		22	Bonferroni Threshold		23	Bonferroni Threshold		22	Bonferroni Threshold		22

FDR Threshold — Bonferroni Threshold (*p*-value ≤ 0.05/24 = 0.0021) — Bonferroni & FDR Thresholds

4.1.3 MMSE Associations

Supplementary Table 15. *P*-values and corresponding effect sizes (*d*-values) are reported for ROI associations between MMSE and dMRI indices across all ADNI3 participants pooled together (N = 315). For each test the ROIs are ordered by *d*-value. Regions that were significant after correcting for multiple comparisons (FDR q = 0.05) are delineated by a dotted line.

MMSE

FA^{DTI}			FA^{TDF}			AxD^{DTI}			MD^{DTI}			RD^{DTI}		
ROI	*p*-value	*d*-value	ROI	*p*-value	*d*-value	ROI	*p*-value	*d*-value	ROI	p-value	*d*-value	ROI	*p*-value	*d*-value
Fx/ST	3.58E-7	0.64	UNC	3.50E-18	1.15	CGH	7.87E-19	-1.18	UNC	2.48E-18	-1.16	UNC	8.63E-18	-1.14
CGH	2.17E-6	0.60	CGH	6.99E-17	1.10	UNC	7.09E-18	-1.14	CGH	4.69E-18	-1.15	CGH	7.39E-17	-1.10
PTR	3.53E-6	0.58	SS	2.15E-11	0.86	SS	1.03E-13	-0.97	SS	3.84E-14	-0.99	SS	2.21E-13	-0.95
GCC	4.60E-6	0.58	Fx/ST	1.35E-9	0.77	Full WM	7.01E-10	-0.79	CGC	3.04E-12	-0.90	CGC	4.07E-12	-0.89
TAP	4.70E-6	0.58	Full WM	3.46E-9	0.75	Fx/ST	1.19E-9	-0.78	Full WM	6.79E-10	-0.79	Full WM	1.13E-9	-0.78
CC	9.58E-6	0.56	PTR	1.51E-8	0.72	CC	9.30E-9	-0.73	IFO	1.27E-9	-0.77	IFO	1.18E-9	-0.78
Full WM	1.07E-5	0.55	CC	1.57E-8	0.72	GCC	1.41E-8	-0.72	GCC	1.32E-9	-0.77	GCC	3.08E-9	-0.76
BCC	1.91E-5	0.54	CGC	2.13E-8	0.71	CGC	5.22E-8	-0.69	Fx/ST	2.69E-9	-0.76	Fx/ST	4.45E-9	-0.75
IFO	2.01E-5	0.53	GCC	7.55E-8	0.68	BCC	6.44E-8	-0.68	SLF	2.24E-8	-0.71	PTR	1.17E-7	-0.67
CGC	3.07E-5	0.52	BCC	7.97E-8	0.68	SLF	3.72E-7	-0.64	PTR	1.23E-7	-0.67	SLF	3.05E-7	-0.65
SS	1.26E-4	0.48	SCC	1.16E-7	0.67	SCC	4.17E-7	-0.64	CC	1.60E-7	-0.66	CP	5.52E-7	-0.63
SCC	1.38E-4	0.48	IFO	1.66E-7	0.66	IFO	5.08E-7	-0.63	BCC	4.31E-7	-0.64	CC	7.45E-7	-0.62
UNC	2.53E-4	0.46	CP	8.64E-7	0.62	PTR	6.12E-7	-0.63	CP	5.95E-7	-0.63	BCC	1.51E-6	-0.61
CP	3.31E-4	0.45	SLF	1.03E-6	0.62	ACR	3.32E-6	-0.58	ACR	1.32E-6	-0.61	ACR	1.78E-6	-0.60
SFO	1.19E-3	0.40	SFO	7.97E-6	0.56	RLIC	5.61E-6	-0.57	RLIC	1.57E-6	-0.60	TAP	1.87E-6	-0.60
ACR	3.94E-3	0.36	TAP	3.67E-5	0.52	PCR	7.46E-6	-0.56	TAP	3.11E-6	-0.59	RLIC	2.47E-6	-0.59
CST	9.17E-3	0.32	RLIC	3.83E-5	0.52	SFO	1.27E-5	-0.55	SFO	1.14E-5	-0.55	SFO	1.23E-5	-0.55
SLF	0.020	0.29	SCR	7.00E-5	0.50	SCR	1.48E-5	-0.54	SCC	1.23E-5	-0.55	SCC	5.45E-5	-0.50
ALIC	0.023	0.28	ACR	1.18E-4	0.48	TAP	1.57E-5	-0.54	SCR	2.21E-5	-0.53	SCR	5.67E-5	-0.50
RLIC	0.042	0.25	PCR	1.30E-3	0.40	CP	8.50E-5	-0.49	PCR	6.65E-5	-0.50	ALIC	1.46E-4	-0.47
EC	0.091	0.21	EC	2.64E-3	0.37	EC	9.41E-5	-0.49	ALIC	1.07E-4	-0.48	EC	2.84E-4	-0.45
PLIC	0.562	0.07	ALIC	0.012	0.31	ALIC	4.41E-4	-0.44	EC	1.54E-4	-0.47	PCR	8.41E-4	-0.42
SCR	0.692	-0.05	CST	0.023	0.28	PLIC	5.73E-4	-0.43	PLIC	6.95E-4	-0.42	PLIC	5.44E-3	-0.35
PCR	0.520	-0.08	PLIC	0.046	0.25	CST	0.150	-0.18	CST	0.086	-0.21	CST	0.071	-0.22
Significant ROIs (N)			Significant ROIs (N)			Significant ROIs (N)			Significant ROIs (N)			Significant ROIs (N)		
FDR Threshold		19	FDR Threshold		24	FDR Threshold		23	FDR Threshold		23	FDR Threshold		23
Bonferroni Threshold		15	Bonferroni Threshold		20	Bonferroni Threshold		23	Bonferroni Threshold		23	Bonferroni Threshold		22

FDR Threshold — Bonferroni Threshold (*p*-value ≤ 0.05/24 = 0.0021) — Bonferroni & FDR Thresholds

4.2. dMRI Associations with Cognitive Measures *by Protocol*

4.2.1 Full WM Clinical Associations

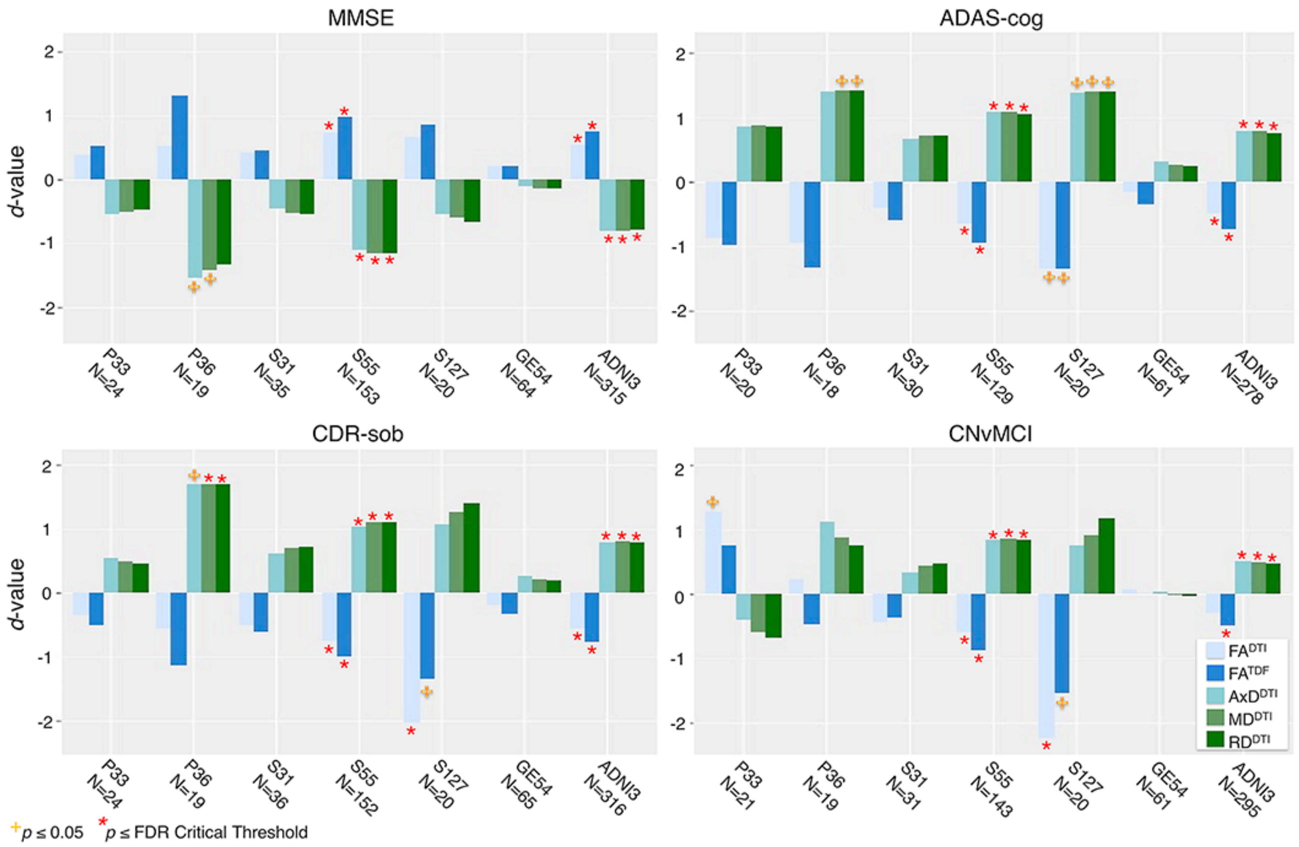

Supplementary Figure 7. For each of six ADNI3 protocols individually and pooled, effect sizes (d-values) are shown for associations between cognitive scores or diagnosis and dMRI indices in the full WM. We note that due to differences in sample size between protocols, effect sizes should not be directly compared. However, the direction of associations was consistent across protocols.

4.2.2 CGH Clinical Associations

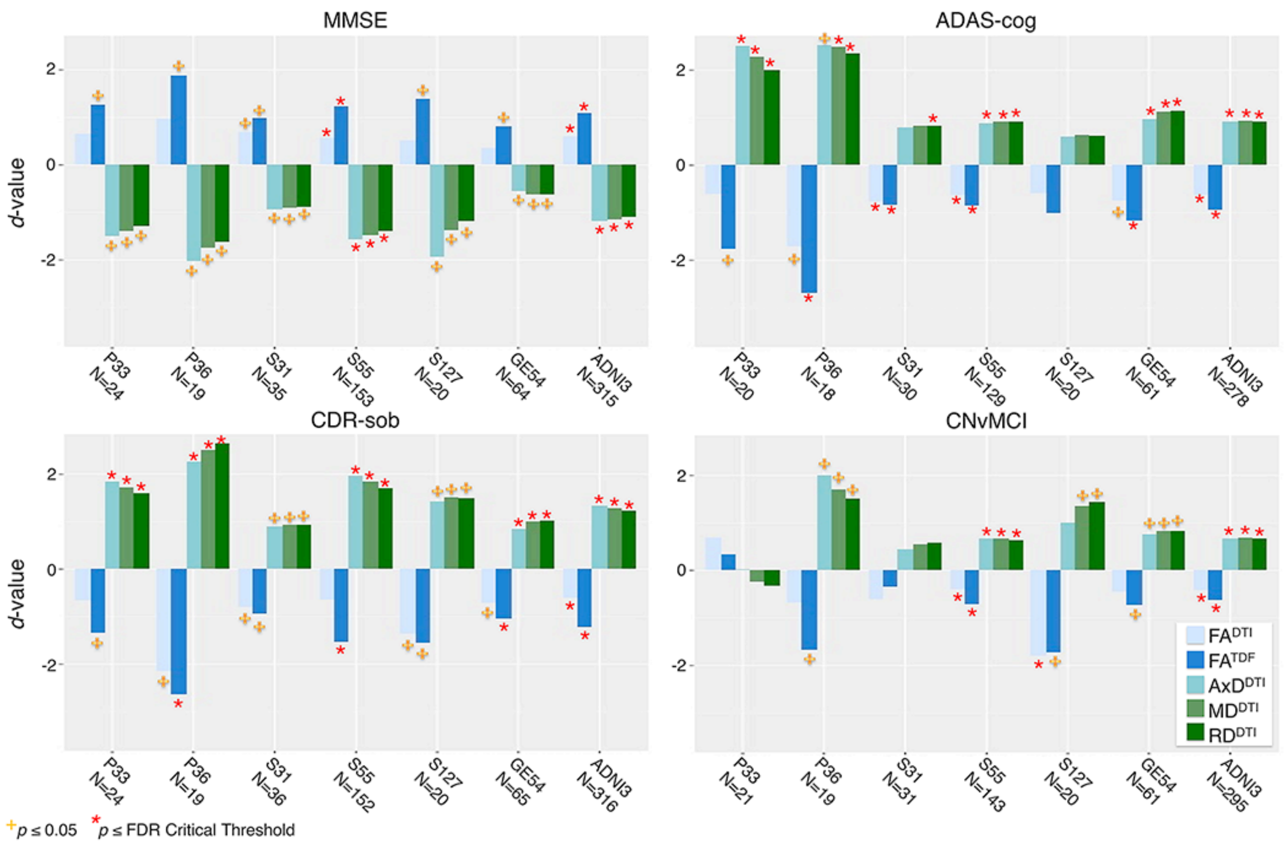

Supplementary Figure 8. For each of six ADNI3 protocols individually and pooled, effect sizes (*d*-values) are shown for associations between cognitive scores or diagnosis and dMRI indices in the CGH – one of two ROIs that consistently showed significant associations across all four clinical tests and dMRI indices. We note that due to differences in sample size between protocols, effect sizes should not be directly compared. However, the direction of associations was consistent across protocols.

4.2.3 Fx/ST Clinical Associations

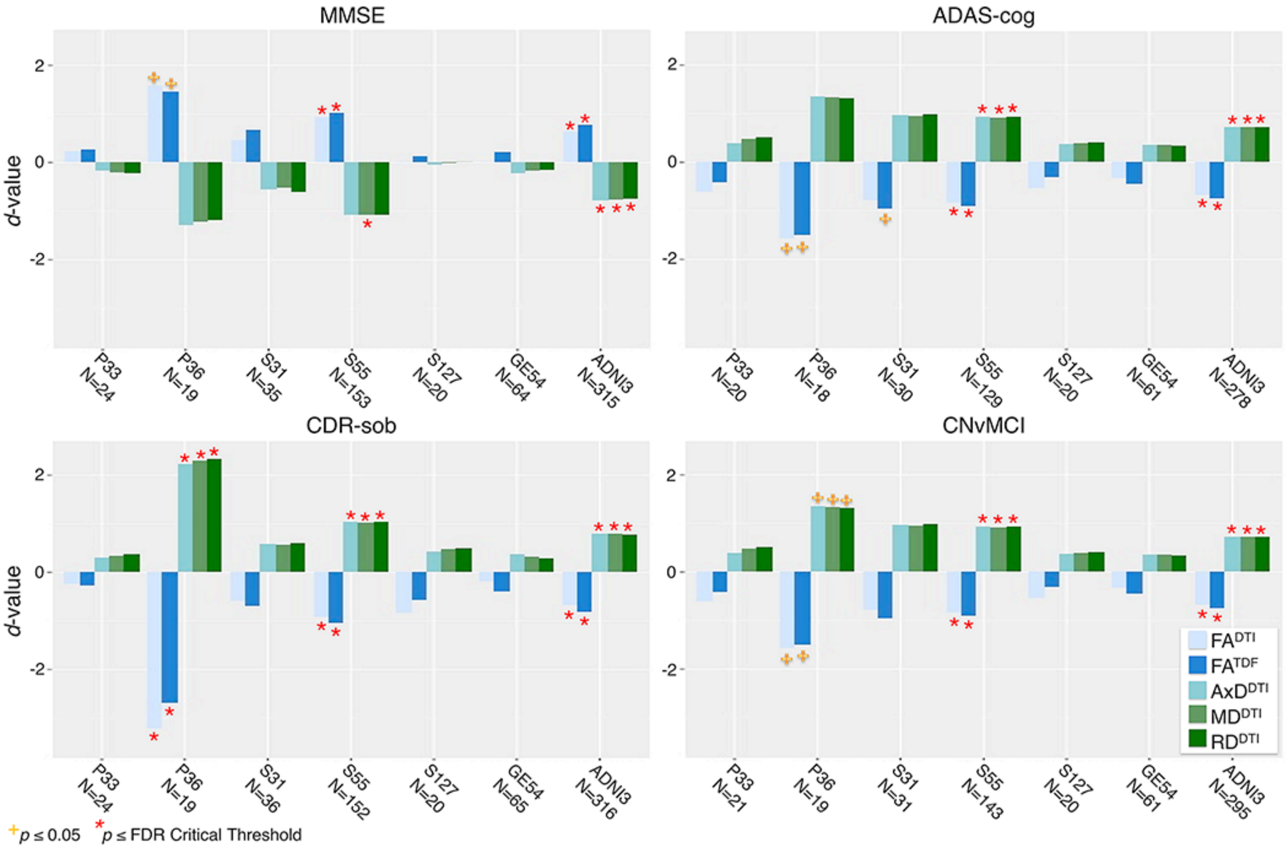

Supplementary Figure 9. For each of six ADNI3 protocols individually and pooled, effect sizes (*d*-values) are shown for associations between cognitive scores or diagnosis and dMRI indices in the Fx/ST – one of two ROIs that consistently showed significant associations across all four clinical tests and dMRI indices. We note that due to differences in sample size between protocols, effect sizes should not be directly compared. However, the direction of associations was consistent across protocols.

4.2.4 FA^{DTI} vs FA^{TDF} Associations with CDR-sob

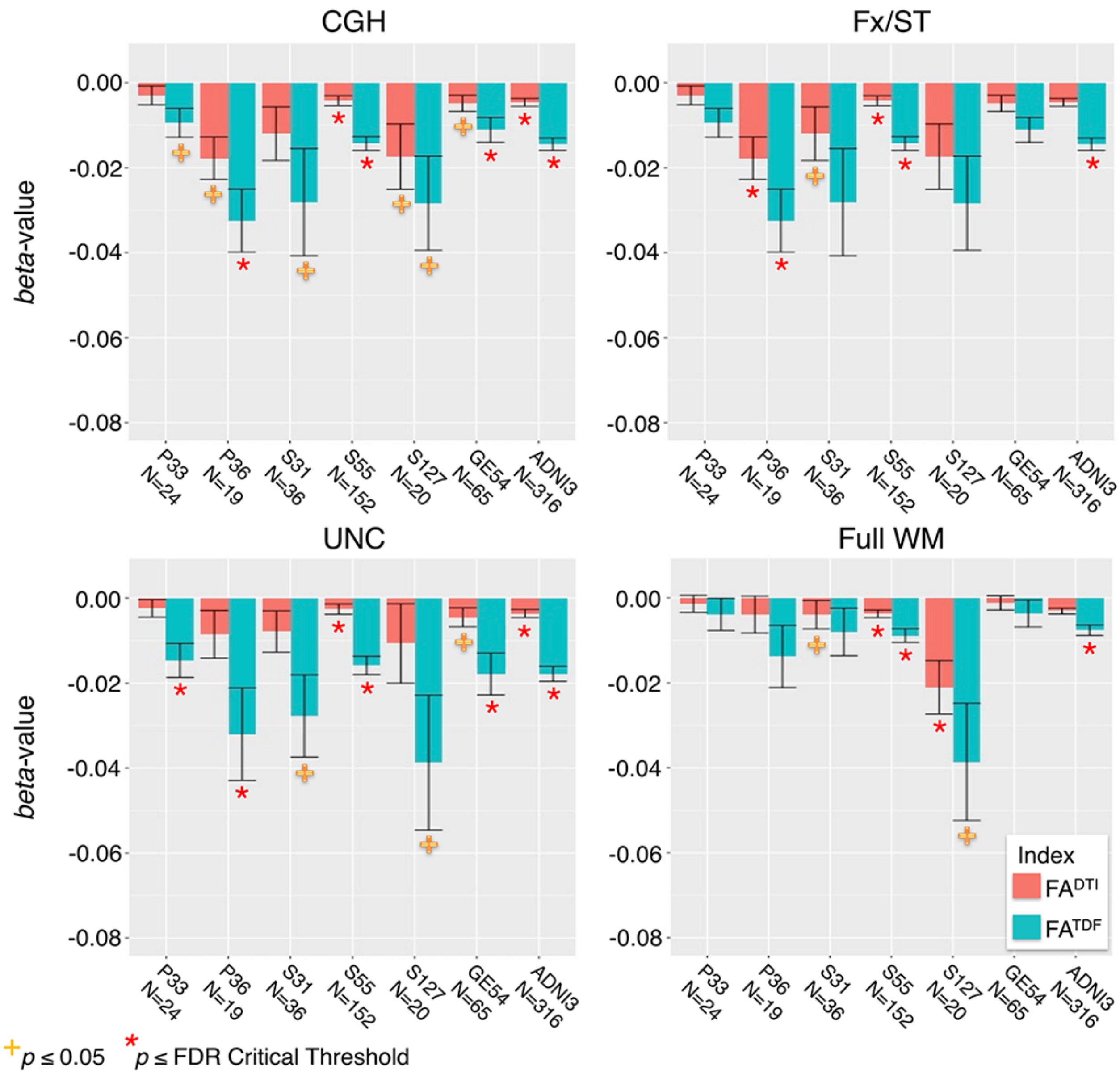

Supplementary Figure 10. A comparison of FA^{DTI} and FA^{TDF} effect sizes (*beta*-values and standard error) for CDR-sob associations in the CGH, Fx/ST, UNC, and full WM, for each of six ADNI3 protocols individually and pooled; FA^{TDF} consistently detected larger effect sizes across protocols.

4.3 Brain Maps of *Pooled* dMRI Associations with Cognitive Measures

4.3.1 ADAS-cog Associations

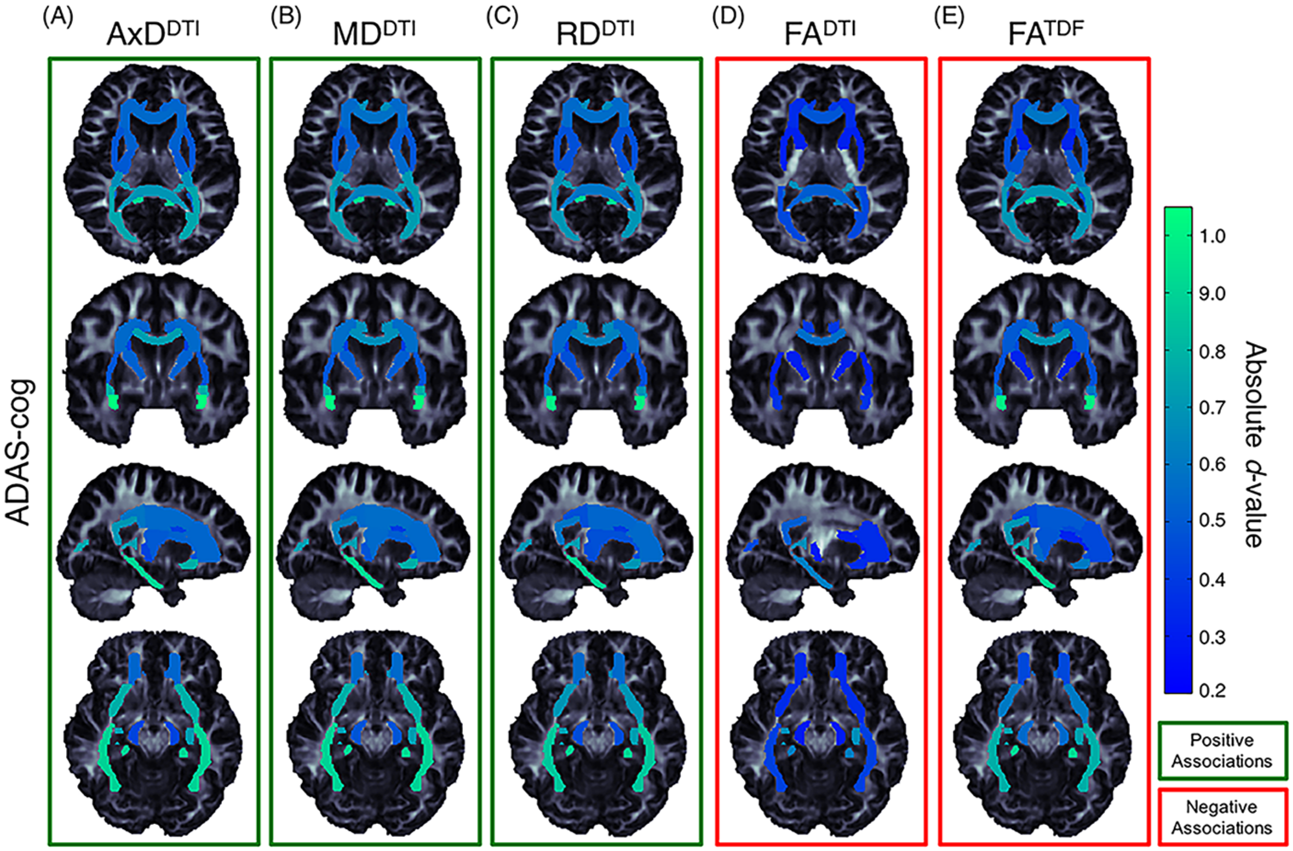

Supplementary Figure 11. Effect size (absolute *d*-value) maps of WM regions that showed significant associations with ADAS-cog (FDR q = 0.05). Associations were detected between ADAS-cog and **(A)** AxD^{DTI}, **(B)** MD^{DTI}, and **(C)** RD^{DTI}, where higher diffusivity was significantly associated with greater cognitive impairment. Lower **(D)** FA^{DTI} and **(E)** FA^{TDF} were associated with greater impairment, but FA^{DTI} associations were detected in fewer regions with weaker effect sizes compared to FA^{TDF}. *Light green* regions show the largest effect sizes.

4.3.2 MMSE Associations

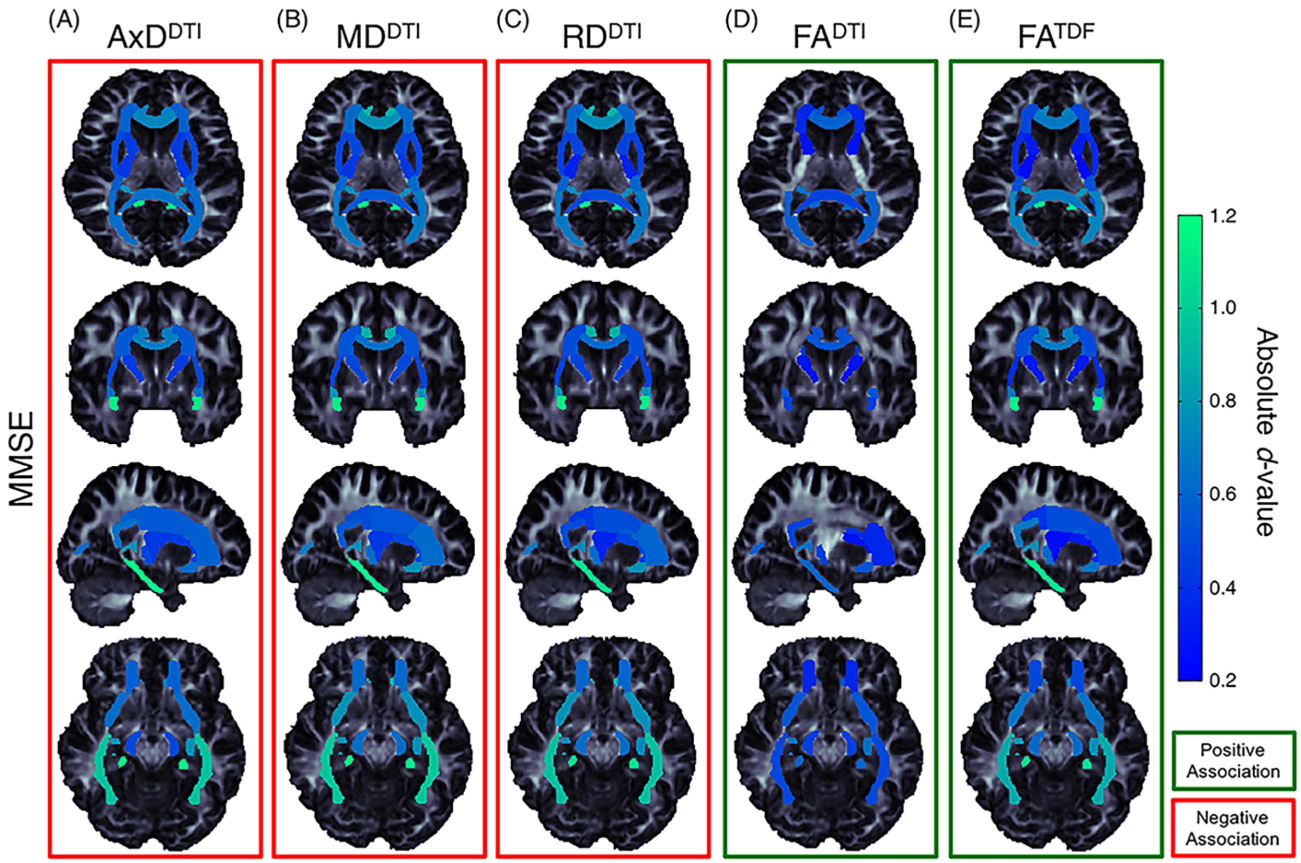

Supplementary Figure 12. Effect size (absolute *d*-value) maps of WM regions that showed significant associations with MMSE (FDR $q = 0.05$). Negative associations were detected between MMSE scores and **(A)** AxD^{DTI}, **(B)** MD^{DTI}, and **(C)** RD^{DTI}, where higher diffusivity was associated with lower MMSE (greater cognitive impairment). Lower **(D)** FA^{DTI} and **(E)** FA^{TDF} were associated with lower MMSE, but FA^{DTI} associations were detected in fewer regions with weaker effect sizes compared to FA^{TDF}. *Light green* regions show the largest effect sizes.

4.4 ROI Size vs CDR-sob Effect Size

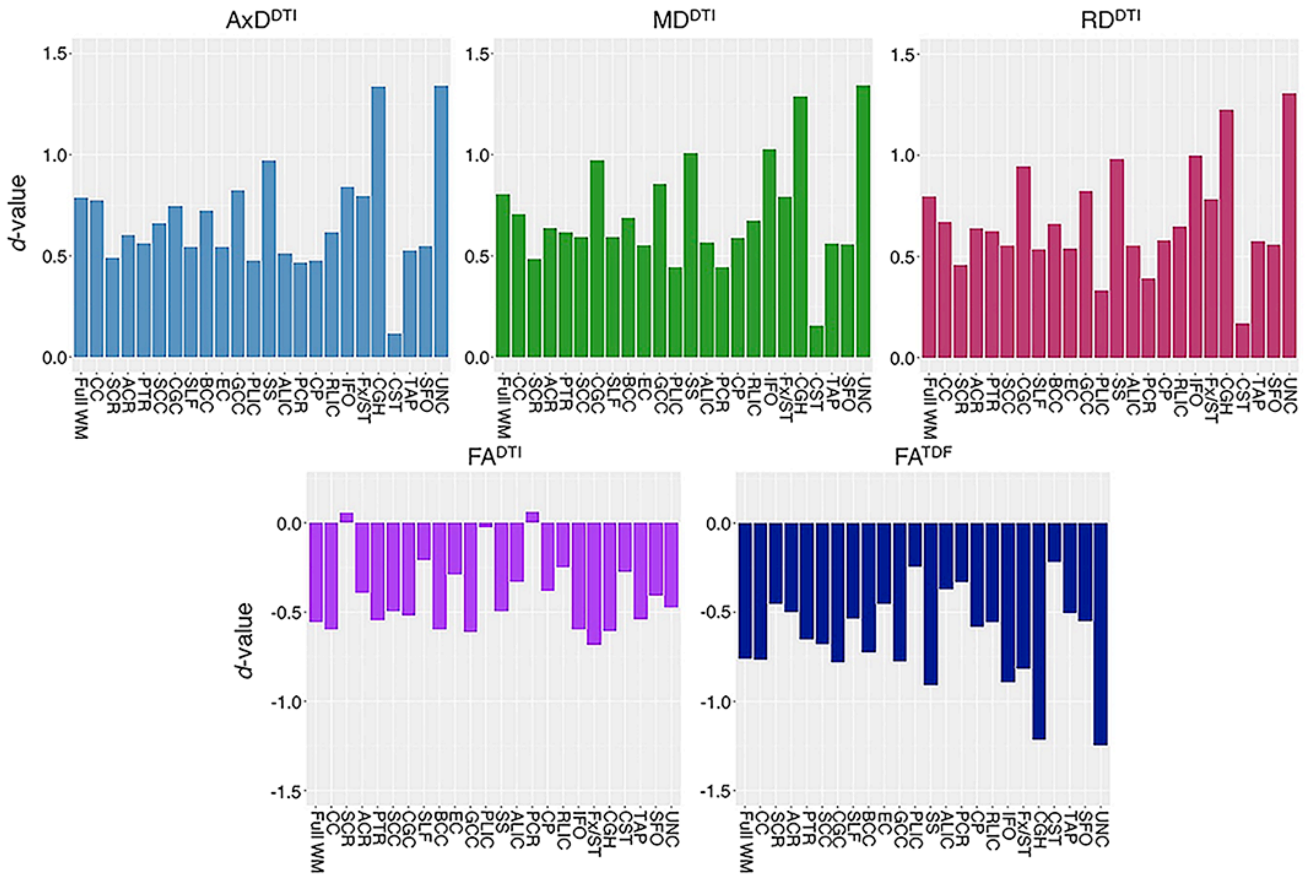

Supplementary Figure 13. CDR-sob effect sizes (*d*-values) for each ROI are ordered by ROI size (full WM, far left, is the largest ROI and UNC, far right, is the smallest). Effect size and the square root of ROI size (number of voxels) were not correlated (FA^{DTI}: Pearson's r = -1.1, p = 0.58; AxD^{DTI}: r = 0.064, p = 0.77; MD^{DTI}: r = 0.044, p = 0.84; RD^{DTI}: r = -0.19, p = 0.93; FA^{TDF}: r = -0.072, p = 0.74).

4.5 Regional dMRI Measures: *Pooled* Associations with Diagnosis

Supplementary Table 16. *P*-values and corresponding effect sizes (*d*-values) are reported for WM microstructural differences between CN and MCI participants when all ADNI3 dMRI data are pooled together. For each test, the ROIs are ordered by *d*-value. Regions that were significant after FDR (q = 0.05) or Bonferroni (α = 0.05) multiple comparisons correction are delineated by a dotted or solid line respectively.

CNvMCI

FA^{DTI}

ROI	*p*-value	*d*-value
Fx/ST	3.89E-4	-0.46
CGH	1.53E-3	-0.41
PTR	4.55E-3	-0.37
TAP	0.012	-0.32
CC	0.018	-0.30
Full WM	0.025	-0.29
GCC	0.029	-0.28
SS	0.033	-0.27
BCC	0.033	-0.27
SCC	0.035	-0.27
SFO	0.054	-0.25
ACR	0.069	-0.23
CP	0.078	-0.23
IFO	0.147	-0.19
EC	0.159	-0.18
RLIC	0.179	-0.17
CST	0.190	-0.17
ALIC	0.207	-0.16
UNC	0.267	-0.14
PLIC	0.351	-0.12
CGC	0.482	-0.09
SLF	0.855	-0.02
PCR	0.858	0.02
SCR	0.823	0.03
Significant ROIs (N)		
FDR Threshold		3
Bonferroni Threshold		2

FA^{TDF}

ROI	*p*-value	*d*-value
CGH	2.00E-6	-0.62
UNC	2.75E-5	-0.55
Fx/ST	4.06E-5	-0.53
SS	7.71E-5	-0.51
Full WM	1.79E-4	-0.49
IFO	2.06E-4	-0.48
PTR	2.06E-4	-0.48
GCC	2.99E-4	-0.47
SFO	5.11E-4	-0.45
CC	5.67E-4	-0.45
SCC	8.00E-4	-0.43
SCR	2.59E-3	-0.39
ACR	4.03E-3	-0.37
BCC	4.11E-3	-0.37
RLIC	4.78E-3	-0.36
TAP	7.27E-3	-0.35
PLIC	7.96E-3	-0.34
PCR	8.09E-3	-0.34
CP	0.022	-0.29
SLF	0.039	-0.27
EC	0.045	-0.26
ALIC	0.056	-0.25
CGC	0.087	-0.22
CST	0.572	-0.07
Significant ROIs (N)		
FDR Threshold		20
Bonferroni Threshold		11

AxD^{DTI}

ROI	*p*-value	*d*-value
CGH	2.26E-7	0.68
UNC	1.58E-6	0.63
IFO	4.77E-6	0.60
GCC	3.17E-5	0.54
SS	3.20E-5	0.54
Full WM	5.58E-5	0.52
Fx/ST	2.24E-4	0.48
ACR	2.87E-4	0.47
CC	3.79E-4	0.46
SFO	4.13E-4	0.46
SCR	4.33E-4	0.46
PTR	5.05E-4	0.45
PCR	1.48E-3	0.41
SCC	1.52E-3	0.41
BCC	2.45E-3	0.39
PLIC	5.86E-3	0.36
RLIC	5.91E-3	0.35
EC	0.012	0.32
TAP	0.029	0.28
ALIC	0.033	0.27
SLF	0.038	0.27
CGC	0.065	0.24
CP	0.078	0.23
CST	0.085	0.02
Significant ROIs (N)		
FDR Threshold		21
Bonferroni Threshold		14

MD^{DTI}

ROI	*p*-value	*d*-value
CGH	1.15E-7	0.70
UNC	5.73E-6	0.59
IFO	8.01E-6	0.58
SS	1.90E-5	0.56
Full WM	1.30E-4	0.50
Fx/ST	2.17E-4	0.48
GCC	2.45E-4	0.48
PTR	2.48E-4	0.48
SCR	5.08E-4	0.45
ACR	5.10E-4	0.45
SFO	5.86E-4	0.45
CC	2.04E-3	0.40
PLIC	2.13E-3	0.40
RLIC	2.58E-3	0.39
PCR	2.96E-3	0.38
BCC	5.22E-3	0.36
SCC	6.68E-3	0.35
EC	0.013	0.32
TAP	0.020	0.30
ALIC	0.025	0.29
CP	0.027	0.28
SLF	0.048	0.25
CGC	0.091	0.22
CST	0.631	0.06
Significant ROIs (N)		
FDR Threshold		21
Bonferroni Threshold		12

RD^{DTI}

ROI	*p*-value	*d*-value
CGH	2.41E-7	0.68
UNC	1.66E-5	0.56
SS	3.47E-5	0.54
IFO	1.25E-4	0.50
Fx/ST	2.27E-4	0.48
Full WM	2.39E-4	0.48
PTR	2.49E-4	0.48
SFO	7.52E-4	0.44
SCR	9.08E-4	0.43
ACR	9.37E-4	0.43
GCC	9.48E-4	0.43
RLIC	3.87E-3	0.37
CC	4.38E-3	0.37
PLIC	6.55E-3	0.35
SCC	7.56E-3	0.34
PCR	8.15E-3	0.34
BCC	8.31E-3	0.34
EC	0.017	0.31
TAP	0.018	0.30
CP	0.028	0.28
ALIC	0.033	0.27
SLF	0.101	0.21
CGC	0.129	0.19
CST	0.550	0.08
Significant ROIs (N)		
FDR Threshold		21
Bonferroni Threshold		11

········ FDR Threshold — Bonferroni Threshold (*p*-value ≤ 0.05/24 = 0.0021) ═ Bonferroni & FDR Thresholds

4.6 Brain Maps of *Pooled* dMRI Associations with Diagnosis

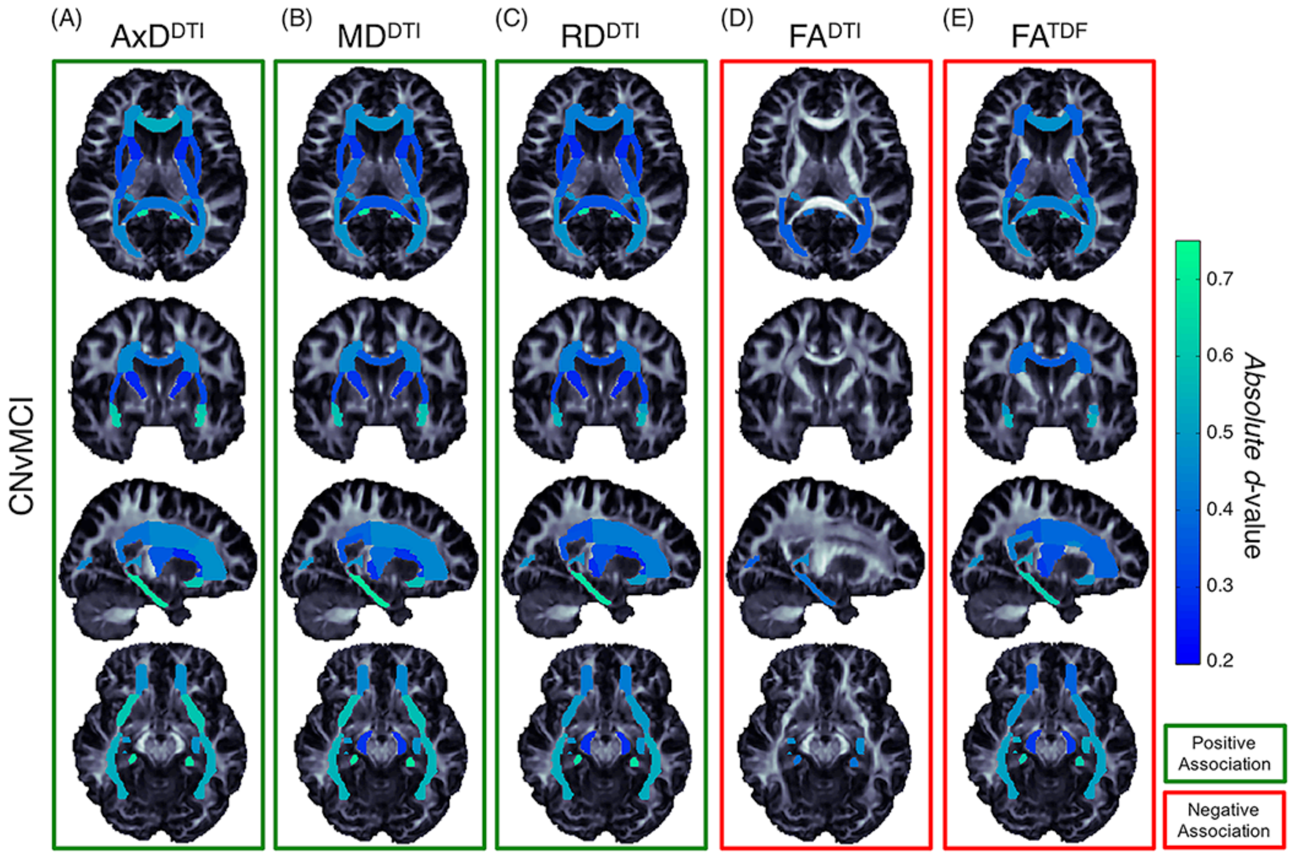

Supplementary Figure 14. Effect size (absolute *d*-value) maps of WM regions that showed significant differences between CN participants and those with MCI (FDR q = 0.05). Positive associations were detected between MCI diagnosis and **(A)** AxD^{DTI}, **(B)** MD^{DTI}, and **(C)** RD^{DTI}, where higher diffusivity was associated with greater cognitive impairment. Lower **(D)** FA^{DTI} and **(E)** FA^{TDF} were associated with greater impairment, but FA^{DTI} associations were detected in fewer regions with weaker effect sizes compared to FA^{TDF}. *Light green* regions show the largest effect sizes.
